# Successional Shifts in Soil C:N:P stoichiometry and its environmental controls during forest restoration in Huize County, Qujing, Yunnan Province

**DOI:** 10.1371/journal.pone.0356261

**Published:** 2026-08-21

**Authors:** Bo Hu, Wei Li

**Affiliations:** 1 College of Soil and Water Conservation, Southwest Forestry University, Kunming, China; 2 Key Laboratory of Ecological Environment Evolution and Pollution Control in Mountainous & Rural Areas of Yunnan Province, Kunming, China; 3 Zhanyi Karst Ecosystem Observation and Research Station, Qujing, China; Shandong University, CHINA

## Abstract

Forest restoration reshapes nutrient cycling and soil stoichiometric regulation in fragile plateau ecosystems. To clarify variations in soil C:N:P stoichiometry and its environmental controls during ecosystem recovery on the southwestern China plateau, this study investigated three representative forest types along a restoration gradient, including Pinus yunnanensis forest, mixed conifer broadleaf forest, and secondary evergreen broadleaved forest. Soil samples were collected from three depths of 0–20 cm, 20–50 cm, and 50–80 cm, together with dominant tree leaf samples, and a total of 27 composite soil samples and 81 undisturbed soil cores were analyzed. Heatmap analysis, Mantel tests, redundancy analysis, and random forest modeling were used to evaluate stoichiometric characteristics and regulatory mechanisms. The results showed that soil organic carbon or total organic carbon and total nitrogen were the dominant factors controlling soil stoichiometry across forest types, whereas the effects of soil layer, iron and aluminum oxides, pH, and nutrient fractions varied with restoration stage. In the Pinus yunnanensis forest, available K and available Al were the primary predictors of C:P, and total nitrogen was the primary predictor of N:P. In the mixed conifer broadleaf forest, total organic carbon became the primary predictor of C:P and C:N ratios, while nitrogen fractions strongly regulated N:P variation. In the evergreen broadleaved forest, C:P was co-regulated by total P, available Al, TOC, and nitrate nitrogen, while N:P was predominantly governed by total P and total N. The explanatory power of environmental variables increased markedly along the restoration gradient, and nutrient heterogeneity in the 0–20 cm soil layer intensified during later restoration stages.Mixed‑effects models confirmed that forest type × depth interactions significantly modulated Al, Fe, and P stoichiometry, corroborating the successional shift from physical to biogeochemical controls. These results indicate that forest restoration progressively strengthens plant-soil nutrient associations and corresponds to a transition from available K/Al and bulk density constraints in the early-stage plantation toward absolute nutrient pools and organo-mineral interactions in the mature forest.

## 1. Introduction

Forest degradation has severely disrupted nutrient cycling and ecosystem stability in the southwestern China plateau [1,2], one of the most ecologically fragile regions in China due to its steep terrain, intense soil erosion, and highly weathered acidic soils [3,4]. In this region, ecological restoration is not only a process of vegetation recovery, but also a reorganization of biogeochemical cycling and soil nutrient regulation [5–7]. Forest restoration alters litter inputs, root allocation, microbial activity, and organic matter accumulation, which are known to influence soil carbon (C), nitrogen (N), and phosphorus (P) dynamics through enhanced nitrogen mineralization, changing phosphorus availability, and strengthened plant–soil relationships [8–10]. Importantly, restoration is a dynamic successional process rather than a static shift in vegetation type [11]. Different restoration stages are often associated with distinct nutrient limitations and regulatory mechanisms, ranging from relatively weak nutrient cycling and low organic matter accumulation in monospecific forests to increasingly complex biological–chemical feedbacks in later successional communities [12–15]. Understanding how soil C:N:P stoichiometric regulation changes along restoration trajectories is therefore essential for clarifying the mechanisms through which forest recovery reconstructs nutrient cycling and ecosystem functioning in fragile plateau ecosystems.

Ecological stoichiometry provides an effective framework for examining nutrient cycling and ecosystem functioning by linking the balance of C, N, and P among plants, soils, and microorganisms [16,17]. Soil C:N:P stoichiometric characteristics are closely associated with nutrient limitation, microbial metabolism, and vegetation succession, and are therefore widely used as indicators of ecosystem development and restoration status [18–20]. In forest ecosystems, SOC and TN generally increase with vegetation recovery a pattern often attributed to enhanced litter and root-derived carbon inputs, whereas TP often remains relatively stable because of strong parent-material constraints [21–23]. Recent global-scale evidence further indicates that phosphorus release from silicate rock weathering can mitigate phosphorus limitation of vegetation growth, with a relative contribution of approximately 15.5% [24].Consequently, shifts in C:P and N:P ratios frequently reflect changes in nutrient availability and ecosystem nutrient limitation [25]. As restoration proceeds, increasingly complex vegetation structure and litter inputs are expected to strengthen vegetation–soil relationships and plant–soil stoichiometric coordination [26].

Previous studies have demonstrated that forest restoration substantially modifies soil nutrient concentrations and stoichiometric ratios through changes in vegetation composition and soil physicochemical properties [27,28]. Surface SOC and TN commonly accumulate during succession, which has been associated with greater litter deposition and fine-root turnover, while upper soil layers exhibit clear nutrient enrichment associated with biological inputs [29–31]. At the same time, shifts in vegetation type alter nutrient acquisition and decomposition pathways, leading to changes in nutrient limitation patterns during restoration [32]. Monospecific forests are often characterized by relatively weak nutrient cycling and are typically associated with low-quality litter inputs, whereas later successional forests exhibit enhanced nitrogen cycling and stronger nutrient turnover [33,34]. Increasing evidence also suggests that restoration promotes tighter coordination between vegetation nutrient traits and soil stoichiometric characteristics [11,35,36]. However, the existing literature has three major limitations. First, most studies have focused on either a single forest type or discrete restoration stages, and have rarely examined the continuous evolution of stoichiometric regulatory mechanisms along a complete successional gradient [11,37]. Second, although Fe and Al (hydr)oxides and pH are known to modulate P sorption and organic matter stabilization in acidic soils [38–41], their changing roles across successional stages—and how they interact with carbon and nitrogen fractions—remain poorly quantified. Third, while plant–soil nutrient coordination is presumed to strengthen with restoration, direct multivariate evidence for the transition from physically constrained (e.g., depth-dependent) to biologically and chemically mediated controls is still lacking [42,43]. Quantitative assessments of these multi-factor interactions across representative successional stages remain scarce, and further investigation is warranted.

In the study region, previous phytosociological studies have demonstrated that secondary succession following anthropogenic disturbance typically follows a predictable trajectory from pioneer Pinus yunnanensis forests, through mixed conifer–broadleaf transitional communities, towards the zonal semi-humid evergreen broadleaved forests [44]. This well-documented successional framework provides a robust and ideal chronosequence for examining how plant–soil nutrient associations and soil stoichiometric regulation evolve during ecosystem recovery. Moreover, the successional classification of tree species utilized in local restoration trials further corroborates the functional differentiation of these seral stages, reinforcing their suitability for such analysis [45]. Along this restoration gradient, increasing vegetation heterogeneity modifies litter deposition, root distribution, soil moisture, and microbial activity, which in turn contributes to observed patterns of nutrient enrichment and heterogeneity in surface soils [46,47], while deeper layers remain more strongly constrained by parent material [48].Based on this framework, the present study investigated soil C:N:P stoichiometric characteristics and their environmental controls across different restoration stages on the southwestern China plateau. Specifically, this study addressed two key scientific questions: (1) How does forest restoration reshape the coordination relationships between plant nutrient characteristics and soil C:N:P stoichiometry across successional stages, and does ecosystem recovery lead to progressively stronger plant–soil biogeochemical coordination? (2) How do the dominant regulatory mechanisms of soil stoichiometry transition during restoration, particularly with respect to the relative roles of vertical stratification, SOC accumulation, nitrogen transformation, pH regulation, and Fe/Al-mediated phosphorus interactions in controlling nutrient balance and ecosystem nutrient limitation?

The innovations of this study are twofold. First, it systematically assesses soil stoichiometric regulation along a complete successional gradient under uniform environmental background, a context rarely examined in this region. Second, by integrating Mantel tests, RDA, and random forest, it quantifies the shifting importance of depth, carbon, nitrogen, Fe/Al oxides, and pH, revealing a transition from depth-dominated to nitrogen- and pH-mediated controls.

## 2. Materials and methods

### 2.1. Profile of the study area

**Fig 1:**
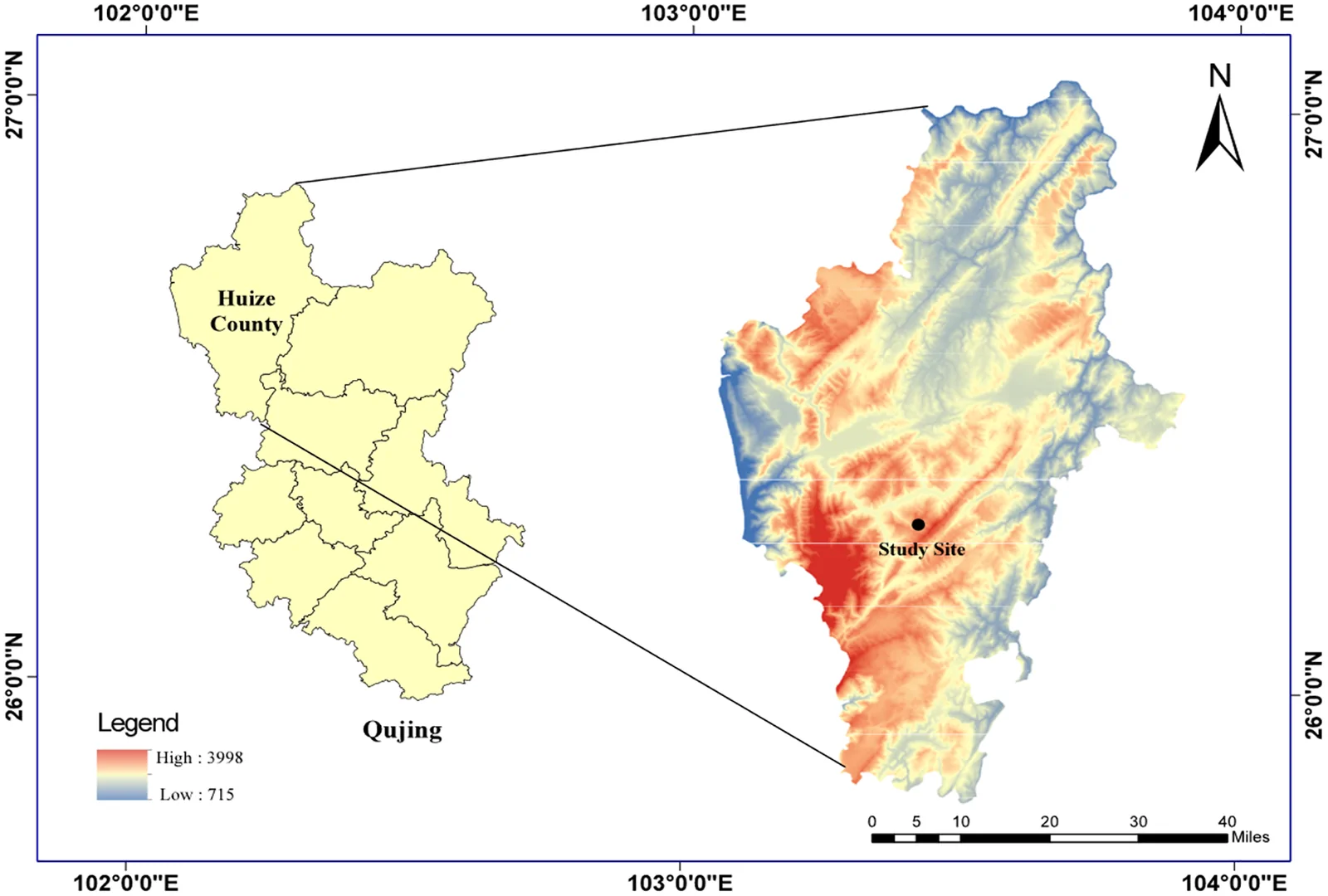
Location of the study area in Huize County, Qujing City, Yunnan Province, China. The data set is provided by Geospatial Data Cloud site, Computer Network Information Center, Chinese Academy of Sciences(http://www.gscloud.cn).

The study area is located in Fengzijing Village, Huize County, Qujing City, Yunnan Province, at the southern foothills of the main peak of the Wumeng Mountains in northeastern Yunnan (approximately 103°03′–103°55′ E, 25°48′–27°04′ N). This region belongs to the Jinsha River watershed, with an average elevation of about 2,100 m. It experiences a south temperate monsoon climate, with a mean annual temperature of 12.7 °C, annual sunshine duration of approximately 2,100–2,158 h, and mean annual precipitation of about 807 mm. Owing to the influence of the plateau mountainous topography, the area has a distinct wet–dry seasonal pattern, and most precipitation occurs from April to September. In terms of vegetation zonation, the study area belongs to the subtropical evergreen broadleaved forest region, and the zonal vegetation is semi-humid evergreen broadleaved forest. However, long-term anthropogenic disturbance has resulted in the scarcity of primary vegetation. The current vegetation is dominated by Pinus yunnanensis forest, mixed conifer–broadleaf forest, and secondary evergreen broadleaved forest, representing a typical forest restoration and successional sequence. The main soil types are red soils and brown soils developed from basalt-derived residual and colluvial materials, and the soils are acidic. Historical over-logging and agricultural reclamation have severely degraded the native vegetation and caused pronounced soil degradation. In recent years, ecological restoration measures such as cropland-to-forest conversion and hill closure for afforestation have effectively promoted vegetation recovery, leading to a secondary successional pattern transitioning gradually from coniferous forest to mixed conifer–broadleaf forest and then to evergreen broadleaved forest. In this study, these three representative forest types were selected as the restoration successional sequence, and plots were established within the study area for systematic sampling(Fig 1).

### 2.2. Soil and plant sample collection

#### 2.2.1. Soil sample collection.

Within the study area, three typical vegetation types were selected according to plant community distribution: Pinus yunnanensis forest, mixed conifer–broadleaf forest, and secondary evergreen broadleaf forest. To minimize spatial autocorrelation, three large sampling regions were established, with a minimum distance of >3 km between regions. In each region, one 20 m × 20 m plot was established for each community type, and the distance between plots within the same region was > 100 m. In total, nine plots were established, all under the same climatic conditions and soil parent material background.

Within each forest type, to account for micro-topographic heterogeneity, nine spatially independent plots (20 m × 20 m) were established along the slope gradient (three on the upper slope, three on the middle slope, and three on the lower slope), with the distance between adjacent plots exceeding 100 m to minimize spatial autocorrelation. In addition, the three forest types exhibited clear structural gradients consistent with successional progression: the mean stand height and diameter at breast height (DBH) increased progressively from the Pinus yunnanensis forest (height: 10.9 ± 0.9 m; DBH: 87.3 ± 2.8 mm) through the mixed conifer–broadleaf forest (height: 11.1 ± 0.9 m; DBH: 92.5 ± 7.6 mm) to the evergreen broadleaf forest (height: 11.6 ± 0.6 m; DBH: 96.1 ± 10.8 mm), further supporting their designation as distinct restoration stages (detailed stand structural parameters are provided in S1 Table). In each plot, soil samples were collected from three depth intervals: 0–20 cm, 20–50 cm, and 50–80 cm. At each plot, five subsamples were collected using a five-point sampling method and then thoroughly mixed. After homogenization and sieving, the samples were divided into three portions to obtain one composite sample for the determination of basic physicochemical properties and soil carbon fractions. All composite samples were passed through a 2 mm sieve to remove roots and gravel. In addition, three undisturbed soil cores were collected from each soil layer using a metal core ring for the determination of soil bulk density (BD) and soil water content (SWC). Thus, for each plot and soil layer, one composite sample and three undisturbed soil cores were collected, yielding a total of 81 composite soil samples (3 forest types × 9 plots × 3 soil layers) and 243 undisturbed soil cores. It is important to emphasize that the five-point mixing was used only to reduce within-plot micro-variability; the true independent unit for statistical analyses is the plot (n = 9 per forest type), rather than individual subsamples. All soil samples were collected in June 2025, during the peak growing season.

#### 2.2.2. Plant sample collection.

In each plot, 5–10 dominant trees were selected, and fully expanded, healthy current-year mature leaves from the sunlit side of the canopy were collected and mixed to form one plant sample. The plant samples were first killed at 105 °C for 30 min, then oven-dried at 65 °C to constant weight, and finally ground and sieved. All plant samples were collected during the peak growing season (June 2025) and the dry season (November 2025) to ensure comparability of the data.

### 2.3. Measurement indicators and methods

#### 2.3.1. Preliminary treatment of soil samples.

After natural air-drying, the collected composite soil samples were cleared of plant residues and gravel, and then sieved with different mesh sizes for subsequent analyses. Samples passed through a 2 mm sieve were used for the determination of pH, available phosphorus (AP), available potassium (AK), exchangeable active Fe and Al, ammonium nitrogen (NH_4_⁺ -N), and nitrate nitrogen (NO_3_ − -N). Samples passed through a 0.25 mm sieve were used for the determination of soil organic carbon (SOC), total nitrogen (TN), total phosphorus (TP), total aluminum (TAl), and total iron (TFe). Undisturbed soil cores were used to determine soil bulk density (BD) and soil water content (SWC).

#### 2.3.2. Determination of soil physical properties.

Soil bulk density (BD) and soil water content (SWC) were determined using the oven-drying method. Undisturbed soil cores of known volume were dried in an oven at 105 °C to constant weight. Soil water content was calculated from the mass loss before and after drying, and soil bulk density was calculated as the ratio of dry soil mass to ring-core volume.

#### 2.3.3. Determination of soil chemical properties.

Soil pH was measured by the electrode method at a soil-to-water ratio of 2.5:1. Soil organic carbon (SOC) was determined using the potassium dichromate oxidation external heating method [49]. Soil total nitrogen (TN) was determined by the Kjeldahl method [50]. Soil total phosphorus (TP), total aluminum (TAl), and total iron (TFe) were measured by inductively coupled plasma atomic emission spectrometry (ICP-AES) after acid digestion with HCl-HNO_3_-HF [51]. Soil available phosphorus (AP) was extracted using the HCl–NH_4_F method and determined by the molybdenum antimony anti-colorimetric method [52]. Soil available potassium (AK) was extracted with ammonium acetate and measured by flame photometry [53]. Soil available iron (AFe) and available aluminum (AAl) were extracted with ammonium acetate, and the extracts were analyzed by ICP-AES [54]. Soil ammonium nitrogen (NH_4_⁺ -N) and nitrate nitrogen (NO_3_− -N) were extracted with 2 mol/L KCl solution at a soil-to-water ratio of 1:10; NH_4_⁺ -N was determined by the indophenol blue colorimetric method, and NO_3_− -N was measured by ultraviolet spectrophotometry [55].

#### 2.3.4. Determination of plant samples.

Plant samples were killed at 105 °C for 30 min, dried at 65 °C to constant weight, and then ground and sieved. Plant total nitrogen (Plant TN) and total phosphorus (Plant TP) were determined after H_2_SO_4_-H_2_O_2_ digestion. The digestion solution was analyzed for total nitrogen using a continuous flow analyzer, and total phosphorus was determined by the vanadium–molybdenum yellow colorimetric method [56]. Leaf carbon-to-nitrogen ratio (C:N) and nitrogen-to-phosphorus ratio (N:P) were calculated from the measured values.

### 2.4. Statistical analysis

The Kolmogorov–Smirnov test (p = 0.05) was used to assess the normality of all variables, and all data are presented as the mean ± standard deviation (SD). Pearson correlation analysis was performed to calculate correlation coefficients among variables and to generate heatmaps. Mantel tests (999 permutations) were further conducted to evaluate the relationships between the distance matrices of soil C:N, C:P, and N:P ratios and those of environmental factors, including pH, bulk density, water content, iron and aluminum oxides, nitrogen forms, and plant N:P ratio. Redundancy analysis (RDA) was used to determine the extent to which environmental factors explained variation in soil C:N:P stoichiometric characteristics. Prior to RDA, detrended correspondence analysis (DCA) was performed, and because the length of the first axis was < 3.0, RDA was selected. Soil C:N, C:P, and N:P ratios were used as response variables, while soil physicochemical properties (BD, SWC, pH, SOC, TN, TP, AP, AK, AFe, AAl, TFe, TAl, NH_4_⁺ -N, and NO_3_− -N) and plant N:P ratio were used as explanatory variables. The significance of the RDA was verified using Monte Carlo permutation tests. A random forest model (ntree = 500, mtry = one-third of the number of predictor variables) was applied to quantitatively evaluate the relative importance of each environmental factor. Variable importance was assessed using %IncMSE and IncNodePurity, and significant predictors were identified by permutation tests (p < 0.05). All statistical analyses and figures were performed using R software (version 4.3.2) and the relevant packages (vegan, randomForest, and ggplot2).Linear mixed‑effects models (lme4) with forest type, depth, and their interaction as fixed effects and region/plot as random intercepts were used to test interactive effects, with significance assessed via lmerTest and emmeans for post‑hoc comparisons.

## 3. Results

### 3.1. Correlation patterns between soil properties and stoichiometric ratios

**Fig 2:**
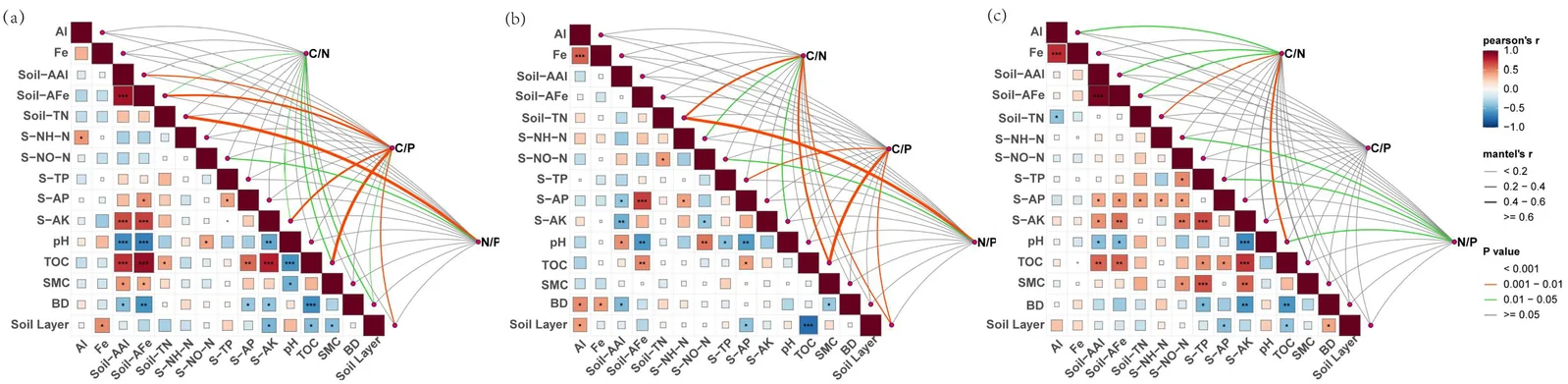
Overall Mantel and Pearson correlation analysis of soil properties and soil C:N:P stoichiometric ratios in Pinus yunnanensis forest(a), mixed conifer–broadleaf forest(b), and evergreen broadleaf forest(c). Red and blue squares indicate positive and negative correlations, respectively, and color intensity reflects the magnitude of Pearson’s r. Asterisks indicate statistical significance, with p < 0.05, p < 0.01, and p < 0.001. The curved lines represent Mantel test results linking environmental variables with soil C:N, C:P, and N:P ratios. Line thickness denotes the strength of the Mantel correlation coefficient. Line colors indicate significance levels: gray, p ≥ 0.05; green, 0.01 ≤ p < 0.05; orange, 0.001 ≤ p < 0.01; and red, p < 0.001. Al, total aluminum; Fe, total iron; Soil-AAl, available aluminum; Soil-AFe, available iron; Soil-TN, total nitrogen; S-NH_4_⁺ -N, soil ammonium nitrogen; S-NO_3_− -N, soil nitrate nitrogen; S-TP, soil total phosphorus; S-AP, soil available phosphorus; S-AK, soil available potassium; pH, soil pH; TOC, total organic carbon; SMC, soil moisture content; BD, bulk density.

Heatmap analysis and Mantel tests revealed distinct patterns of nutrient cycling and environmental regulation across vegetation types. In the Pinus yunnanensis forest(Fig 2a), total organic carbon (TOC) was strongly positively correlated with available Fe (Soil-AFe, r = 0.878, p < 0.001), available K (s-AK, r = 0.783, p < 0.001), and available Al (Soil-AAl, r = 0.730, p < 0.001), indicating that organic carbon accumulation enhances metal ion availability. Soil pH was negatively correlated with Soil-AFe (r = −0.685, p < 0.001) and Soil-AAl (r = −0.631, p < 0.001), while bulk density (BD) declined with TOC (r = −0.618, p < 0.001), suggesting that compaction inhibits organic matter storage. Mantel tests showed that the C/N ratio increased with TOC and BD, the C/P ratio was mainly driven by TOC (r = 0.666, p = 0.001), s-AK, and Soil-AFe, and the N/P ratio was influenced by total N and nitrate nitrogen.

In the mixed conifer–broadleaf forest(Fig 2b), total Al and total Fe were strongly coupled (r = 0.600, p < 0.001), and available P increased with available Fe (r = 0.695, p < 0.001). TOC declined with soil depth (r = −0.764, p < 0.001), reflecting surface enrichment. Unlike P. yunnanensis, pH was positively associated with available Al (r = 0.460, p = 0.016). Mantel tests indicated that C/N was enhanced by TOC, total N, and available K; C/P was driven predominantly by TOC (r = 0.727, p = 0.001); and N/P increased with total N (r = 0.685, p = 0.001) and nitrate nitrogen.

In the evergreen broadleaf forest(Fig 2c), available Al and Fe were tightly coupled (r = 0.953, p < 0.001). SOC exhibited broad positive correlations with available Al, Fe, and K (r = 0.46–0.72). Available K increased with total P (r = 0.665) and SOC (r = 0.715, p < 0.001) but decreased with pH (r = −0.641). Mantel tests showed that C/N was primarily driven by pH (r = 0.505, p = 0.001), N/P was influenced by total P, pH, and ammonium nitrogen, and C/P showed no significant correlations.

Overall, SOC (or TOC) and total N emerged as the core drivers of soil C:N:P stoichiometry across forest types, while iron and aluminum oxides and pH played important auxiliary roles in modulating nutrient availability, with the strength and direction of their effects varying among ecosystems.

### 3.2. Successional shifts in regulatory mechanisms of soil C:N:P stoichiometry

**Fig 3:**
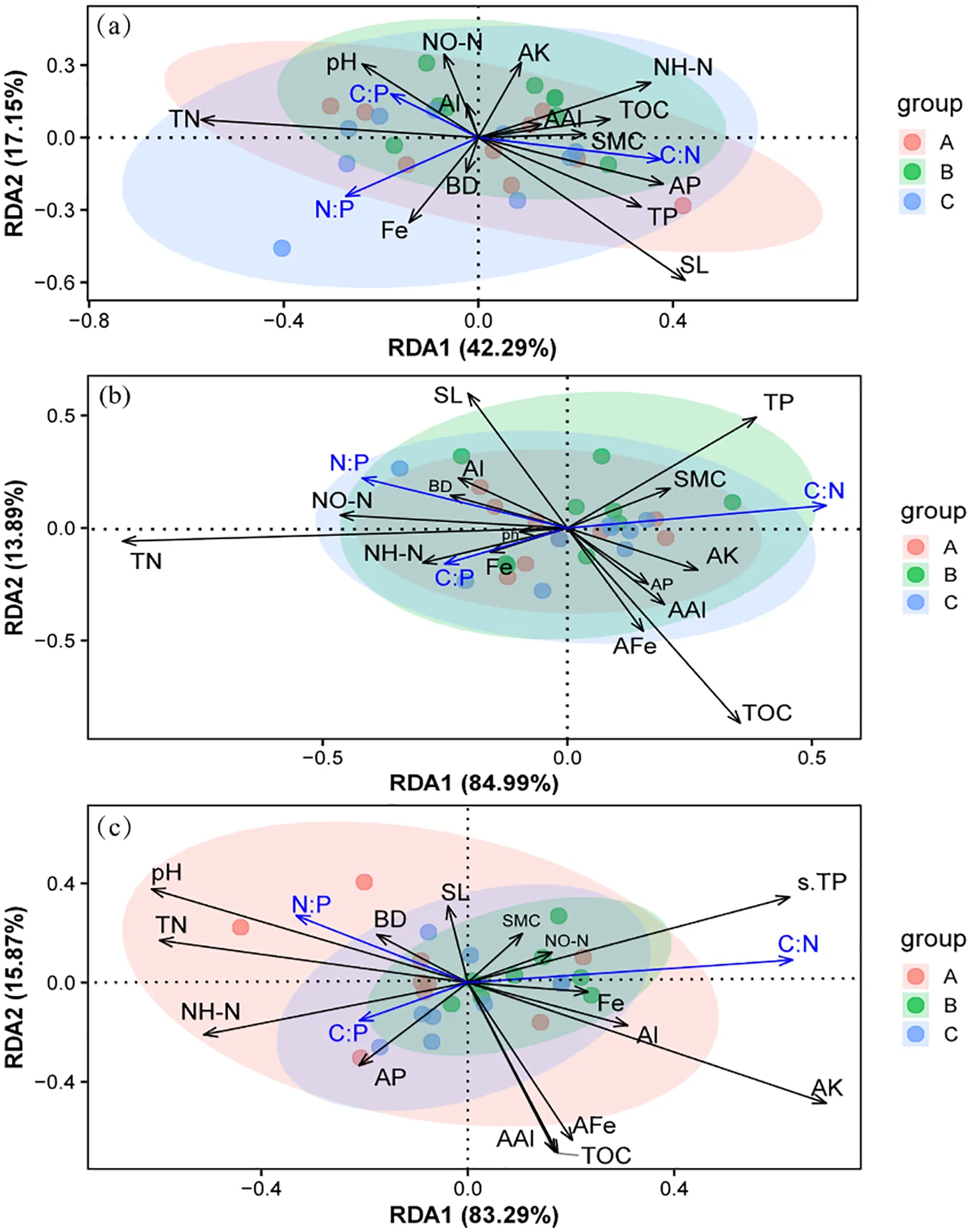
Redundancy analysis (RDA) of soil C:N:P stoichiometric ratios and environmental variables in Pinus yunnanensis forest(a), mixed conifer–broadleaf forest(b), and evergreen broadleaf forest(c). Blue arrows indicate soil stoichiometric ratios (C:N, C:P, and N:P), and black arrows indicate environmental variables. Arrow length reflects the strength of the relationship, and arrow direction indicates the correlation pattern. The colored ellipses represent the three sample groups (A, B, and C). pH, soil pH; TOC, total organic carbon; SMC, soil moisture content; BD, bulk density; SL, soil layer; TN, soil total nitrogen; TP, soil total phosphorus; AP, soil available phosphorus; AK, soil available potassium; AFe, soil available iron; AAl, soil available aluminum; NH_4_⁺ -N, soil ammonium nitrogen; NO_3_− -N, soil nitrate nitrogen; C:N, soil C:N; C:P, soil C:P; N:P, soil N:P.

Redundancy analysis (RDA) was performed based on plot-level replicates (n = 9 per forest type). The results revealed a clear successional transition in the regulatory mechanisms of soil C:N:P stoichiometry, characterized by an increase in both model significance and explanatory power as restoration proceeds. While the Pinus yunnanensis forest(Fig 3a) exhibited a non-significant global model (F = 1.080, p = 0.424) with environmental variables explaining only 59.44% of the variance, the more advanced mixed conifer–broadleaf(Fig 3b) and evergreen broadleaf(Fig 3c) stages showed highly significant relationships (p = 0.001), with total explained variation rising to 98.88%–99.16%. A fundamental shift in dominant drivers was observed across this gradient: in the early-stage Pinus forest, soil depth (p = 0.059) and TOC were only marginal regulators, but as the community transitioned toward mixed and evergreen broadleaf forests, nitrogen fractions—particularly Soil-TN (F = 837.6) and s.NH_4^ + -N (F = 384.5)—emerged as the primary predictors. Vertical stratification remained a robust feature across the gradient; however, the dispersion of surface layers (0–20 cm) along the primary axes intensified in later restoration stages, reflecting a greater heterogeneity in nutrient cycling near the surface. Ultimately, these results indicate that ecological restoration shifts soil stoichiometric control from a depth-dependent, carbon-limited system to one complexly regulated by nitrogen availability and phosphorus-pH interactions, highlighting the maturation of nutrient-cycling feedback loops.

### 3.3. Interactive controls of forest type and soil depth on soil stoichiometric characteristics

**Fig 4:**
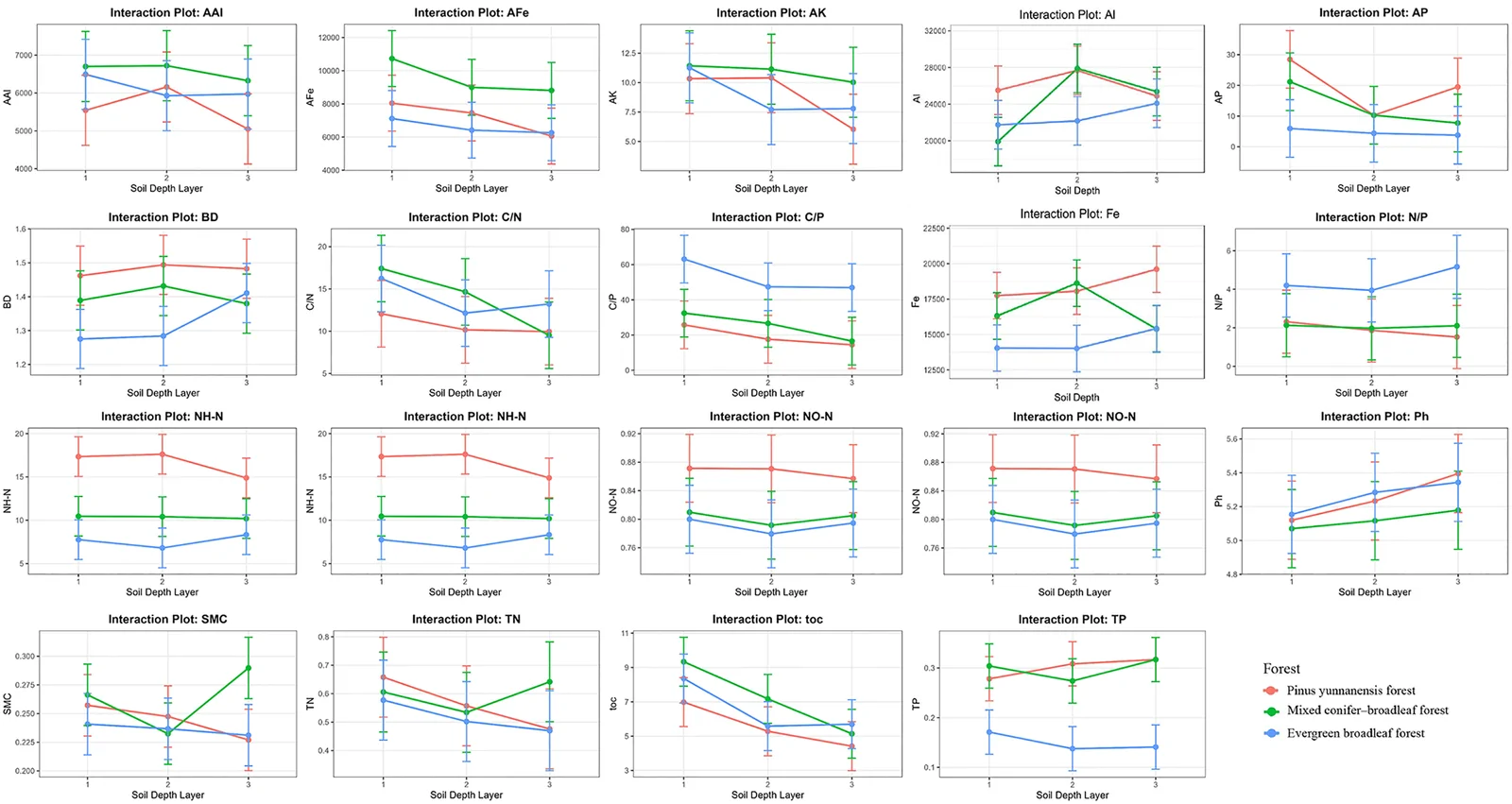
Interactive effects of forest type and soil depth on selected soil properties and stoichiometric ratios. The plots show estimated marginal means (±SD) derived from linear mixed‑effects models, with forest type (Pinus yunnanensis forest, mixed conifer–broadleaf forest, evergreen broadleaf forest) and soil depth layer (1: 0-20, 2: 20-50, 3: 50-80 cm) as fixed effects, and sampling region and plot as random intercepts. Variable abbreviations:TP, soil total phosphorus; TN, soil total nitrogen; AP, soil available phosphorus; AK, soil available potassium; AAl, soil available aluminum; NH_4_⁺ -N, soil ammonium nitrogen; NO_3_− -N, soil nitrate nitrogen; SMC, soil moisture content; BD, bulk density; SL, soil layer; Al, total aluminum; Fe, total iron; C:N, soil C:N ratio; C:P, soil C:P ratio; N:P, soil N:P ratio.

**Fig 5:**
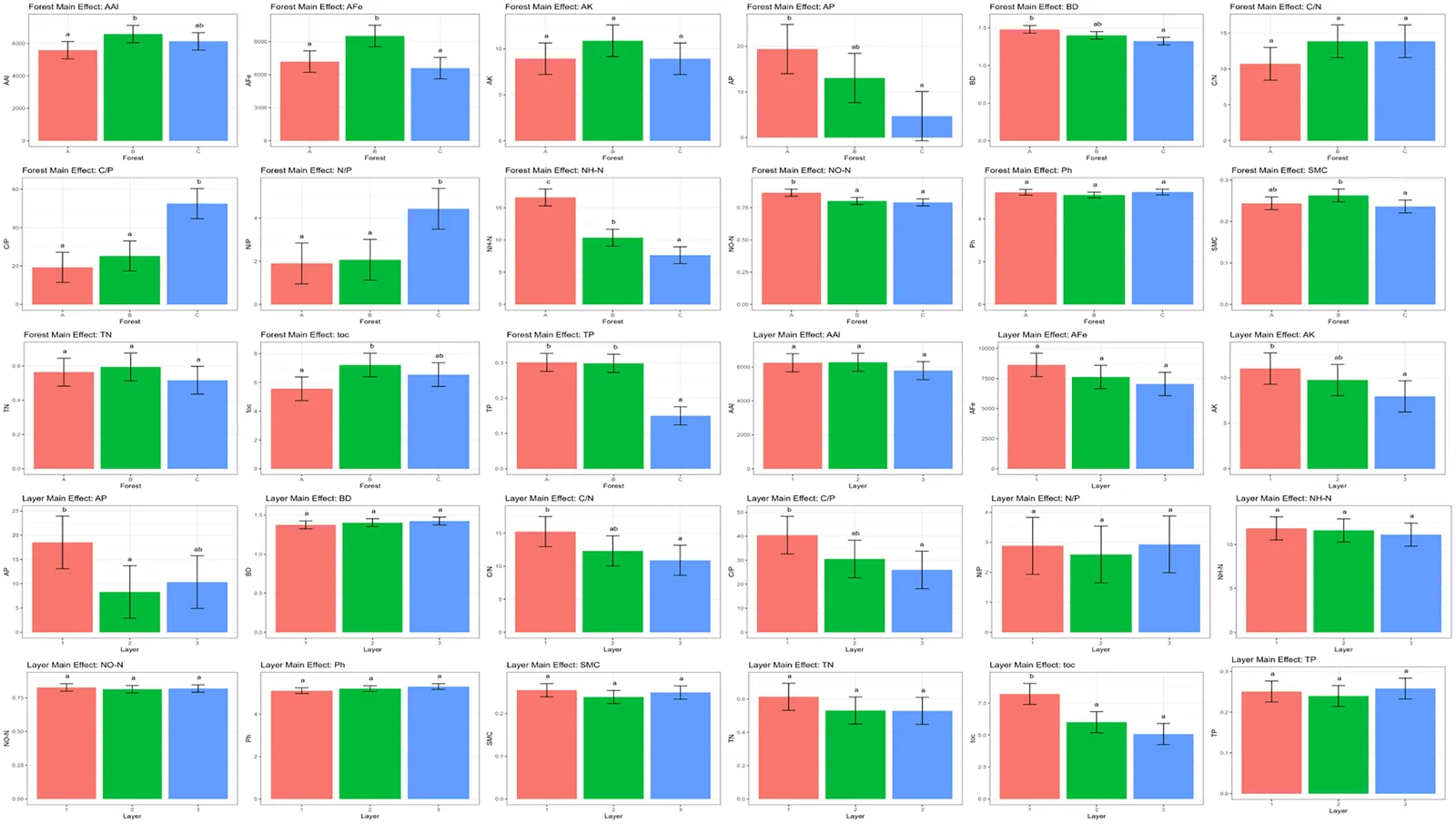
Main effects of forest type on selected soil properties across three depth layers (1: 0–20, 2: 20–50, 3: 50–80 cm). The bar chart shows estimated marginal means (± SD) derived from linear mixed‑effects models, with forest type (A: Pinus yunnanensis forest, B: mixed conifer–broadleaf forest, C: evergreen broadleaf forest) as fixed effect, and sampling region and plot as random intercepts. Different lowercase letters above the bars indicate significant differences among forest types (P < 0.05) based on post‑hoc pairwise comparisons using the emmeans package with Tukey adjustment. Variable abbreviations:TP, soil total phosphorus; TN, soil total nitrogen; AP, soil available phosphorus; AK, soil available potassium; AAl, soil available aluminum; NH_4_⁺ -N, soil ammonium nitrogen; NO_3_− -N, soil nitrate nitrogen; SMC, soil moisture content; BD, bulk density; SL, soil layer; Al, total aluminum; Fe, total iron; C:N, soil C:N ratio; C:P, soil C:P ratio; N:P, soil N:P ratio.

The mixed-effects models revealed distinct effects of forest type, soil depth, and their interaction on soil properties and stoichiometric ratios(Fig 4 and Fig 5). Results showed that BD was higher in P. yunnanensis than in evergreen forest (P < 0.001), with no depth effect. TOC varied with type and depth (both P < 0.05), peaking in mixed forest and declining with depth. TP differed among types (P < 0.001), being elevated in P. yunnanensis and mixed relative to evergreen. NH4 ⁺ -N and NO3 − -N were highest in P. yunnanensis (P < 0.001), but depth and interaction were non‑significant. AP was affected by type and depth (P < 0.05), with maxima in P. yunnanensis and surface layer, whereas AK decreased with depth regardless of type. For stoichiometry, C:N decreased with depth (P < 0.05); C:P was influenced by type and depth (P < 0.05), highest in evergreen and surface; N:P was type‑specific (P < 0.001), higher in evergreen than in the other two. Importantly, significant Forest × Layer interactions were detected for Al and Fe (P < 0.05). For Al, surface concentrations were higher in P. yunnanensis than in mixed; in middle layer, P. yunnanensis and mixed exceeded evergreen; vertical variation occurred only in mixed forest. Fe followed a similar pattern, with P. yunnanensis consistently higher. In contrast, AAl and AFe were type‑dependent (P < 0.05), both peaking in mixed forest. These results confirm that depth effects on stoichiometry are modulated by forest type, particularly for Al/Fe and P‑related ratios.

### 3.4. Key predictors of soil stoichiometric ratios across restoration stages

**Fig 6:**
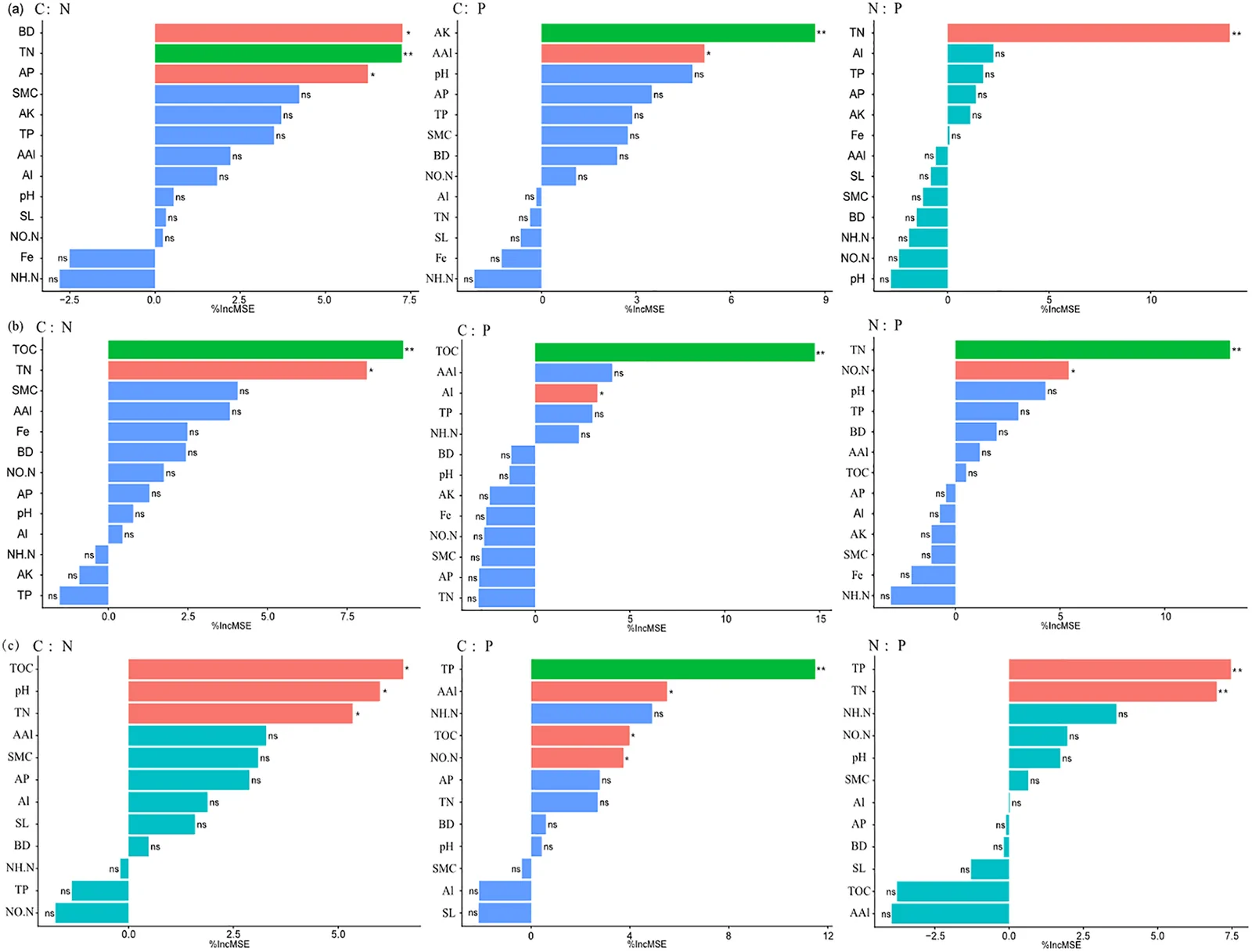
Relative importance of environmental variables in predicting soil C:N:P stoichiometric ratios in Pinus yunnanensis forest(a), mixed conifer–broadleaf forest(b), and evergreen broadleaf forest(c) based on Random Forest analysis. Variable importance was evaluated using the percentage increase in mean squared error (%IncMSE). Positive %IncMSE values indicate that the variable contributes positively to model prediction accuracy, whereas negative values indicate weak or negligible contributions. Asterisks indicate significance levels (* p < 0.05, ** p < 0.01), and “ns” indicates non-significant effects (p ≥ 0.05). TP, soil total phosphorus; TN, soil total nitrogen; AP, soil available phosphorus; AK, soil available potassium; AAl, soil available aluminum; NH_4_⁺ -N, soil ammonium nitrogen; NO_3_− -N, soil nitrate nitrogen; SMC, soil moisture content; BD, bulk density; SL, soil layer; Al, total aluminum; Fe, total iron; C:N, soil C:N ratio; C:P, soil C:P ratio; N:P, soil N:P ratio.

The Random Forest (RF) analysis identified distinct sets of significant predictors for soil C:N:P stoichiometric ratios across the three forest types. In the P. yunnanensis forest(Fig 6a), C:P was primarily predicted by available K (p < 0.01) and available Al (p < 0.05), N:P by total nitrogen (p < 0.01), and C:N by bulk density (p < 0.05), total nitrogen (p < 0.01), and available P (p < 0.05). In the mixed conifer–broadleaf forest(Fig 6b), TOC (p < 0.01) and total nitrogen (p < 0.05) significantly predicted C:N, while C:P was driven by TOC (p < 0.01) and total Al (p < 0.05), and N:P by total nitrogen (p < 0.01) and nitrate nitrogen (p < 0.05). In the evergreen broadleaf forest(Fig 6c), C:P was co-regulated by total P (p < 0.01), available Al (p < 0.05), TOC (p < 0.05), and nitrate nitrogen (p < 0.05); N:P by total P (p < 0.01) and total nitrogen (p < 0.01); and C:N by TOC (p < 0.05), pH (p < 0.05), and total nitrogen (p < 0.05). Overall, the significant drivers shifted from cation-exchange and physical constraints in the pine forest to coupled C–N accumulation and mineral sorption in the mixed forest, and finally to absolute nutrient pool sizes with pH-modulated stabilization in the mature evergreen forest.

## 4. Discussion

### 4.1. Ecological interpretation of correlations between soil properties and stoichiometric ratios

Organic matter regulates P availability in highly acidic soils through the complexation of Fe and Al ions that would otherwise promote the precipitation of insoluble Fe and Al phosphates, thereby reducing P retention [57]. SOC and labile C fractions positively correlated with nutrients (ST, TN, AN, AP, AK) and base cations (Ca, Fe, Al), reflecting enhanced nutrient inputs from decomposed forest floor post-thinning and strong organic matter–mineral interactions governing SOC stability [58,59].In addition, decreasing pH increased the solubility and reactivity of Fe/Al (hydr)oxides, contributing to the negative relationship between pH and Fe/Al (hydr)oxides [60]. Soil compaction alters soil properties by increasing bulk density and reducing porosity, ultimately decreasing SOC content. The decline in SOC with increasing compaction, together with the strong negative correlation between bulk density and SOC, demonstrates that greater compaction restricts organic matter accumulation [61]. Collectively, these patterns highlight strong coupling between soil physicochemical properties and nutrient stoichiometry: carbon availability and bulk density regulate C/N dynamics, C/P is primarily controlled by carbon inputs and nutrient availability, and N/P is closely associated with total and mineral nitrogen fractions [62]. Variations in soil C/N are also closely associated with shifts in soil physicochemical properties and physicochemical stabilization, as coupled carbon and nitrogen pools jointly regulate nutrient cycling and soil quality, whereas SOC and mineral nitrogen influence abiotic sorption-desorption equilibria and nutrient availability, thereby affecting stoichiometric balance [63]. The positive relationships between C/N and SOC and bulk density further support the role of organic carbon accumulation in regulating soil nitrogen dynamics, consistent with evidence that organic mulching enhances soil C and N contents [64]. Similarly, the dependence of C/P on SOC, s-AK, and soil-AFe demonstrates close interactions among phosphorus availability, organic carbon, and soil properties, whereas the influence of total N and nitrate‑N on N/P confirms the importance of nitrogen fractions in regulating soil N availability and stoichiometric balance [65].

In the mixed conifer–broadleaf forest, the strong coupling between total Al and total Fe, together with the positive relationship between available P and available Fe, is consistent with observations that phosphorus sorption capacity is strongly associated with Fe and Al oxide concentrations [66]. The positive relationship between available P and available Fe also confirms the important role of Fe‑containing minerals in regulating P availability through sorption and desorption processes, as amorphous and organically bound Fe oxides were significantly associated with labile P fractions in acidic forest soils [57]. The decline in SOC with increasing soil depth is associated with greater aboveground litter inputs and richer fine‑root biomass in surface soils, both of which promote organic carbon accumulation in upper layers [67]. In contrast, deeper soils receive less fresh organic matter and are subjected to stronger environmental constraints, including lower oxygen availability and poorer substrate quality, thereby limiting carbon stabilization [68]. The positive correlation between pH and available Al in the mixed conifer–broadleaf forest corresponds with increased Al solubility as soil pH approaches 5.5, resulting in elevated exchangeable aluminum concentrations [69]. Simultaneously, tree species traits related to aluminum accumulation may increase soil pH while enhancing aluminum availability through colloid dispersion and the release of occluded cations [70]. The dominant effect of SOC on the C/P ratio demonstrates a strong linkage between organic matter turnover and stabilization and phosphorus availability, whereas the dependence of N/P on total N and nitrate‑N highlights the importance of nitrogen availability following disturbance. Moreover, the enhancement of C/N by SOC, total N, and available K supports asynchronous carbon and nitrogen accumulation during restoration, consistent with reduced grazing promoting carbon accumulation relative to nitrogen [36,71].

In the evergreen broadleaf forest, the tight coupling between available Al and Fe, together with their broad positive correlations with SOC, demonstrates the importance of these metal oxides in SOC stabilization through organo‑mineral complexation. Such chemical protection, potentially mediated by ligand exchange on mineral surfaces, represents an important mechanism of soil carbon accumulation in acidic forest ecosystems [72]. Unlike the potassium depletion reported during karst forest restoration [73], the positive correlations of available K with total P and SOC observed in this study demonstrate that coupled nutrient accumulation and organic matter retention under different lithological or successional conditions can alleviate pH‑induced K limitation. The pH‑driven pattern of soil C/N is partly consistent with observations from afforested systems on the Loess Plateau, where pH was positively associated with C/N [74]. However, other studies have reported either no significant relationship between pH and stoichiometry in alpine marshy wetlands [75]or negative relationships between pH and C/N ratios [76]. These inconsistencies are associated with differences in ecosystem type, restoration stage, and soil depth interval, whereas our acidic, Fe/Al-rich system underscores that mineral-surface sorption serves as the primary differentiated regulatory pathway.

Mechanistically, in contrast to alkaline or calcareous soil systems where stoichiometric ratios are primarily modulated by carbonate buffering and calcium-mediated organic matter stabilization, our acidic, Fe/Al-rich forest soils exhibit a fundamentally different regulatory pathway. Here, the surface sorption and desorption reactions of Fe/Al (hydr)oxides directly govern phosphorus availability and carbon retention, rather than base cation weathering or moisture-limited decomposition. This mineral-surface ligand exchange mechanism constitutes the key differentiated conclusion emerging from our physicochemical data, implying that in humid subtropical acidic forests, the management of organo-mineral complexes may be more critical for nutrient regulation than solely adjusting bulk pH or total nutrient inputs.

### 4.2. Drivers and implications of successional shifts in stoichiometric regulatory mechanisms

The progressive increase in total explained variation from the Pinus yunnanensis forest to the mixed conifer–broadleaf and evergreen broadleaf stages demonstrates a gradual strengthening of plant–soil stoichiometric coordination during restoration. In the early‑stage pure coniferous forest, the dominance of a single tree species with high‑lignin and low‑nutrient litter maintains relatively weak and unstable feedbacks between vegetation and soil, resulting in lower explanatory power of environmental variables. This condition is accompanied by a pronounced stoichiometric imbalance in the surface organic layers [77]. As restoration proceeds toward mixed and evergreen broadleaf forests, the introduction of functionally complementary broadleaf species increases litter diversity and nutrient inputs, improves soil moisture and pH conditions, and enhances the chemical availability of labile C fractions and promotes organo-mineral interactions [78,79]. These changes strengthen bidirectional relationships among vegetation, litter, and soil, while vegetation traits and soil properties exert increasingly direct controls over tissue and soil C:N:P stoichiometry, collectively accounting for nearly all system variance in the later stages. Accordingly, the transition from a relatively decoupled and resource‑limited pine forest to a more integrated and functionally diverse ecosystem corresponds with the marked increase in model significance and explanatory power observed along the restoration gradient.

The successional transition in dominant regulatory factors further demonstrates a shift from physically constrained regulation toward nutrient‑driven biogeochemical control. In the early‑stage Pinus forest, limited organic matter accumulation restricts microbial activity, causing soil depth and SOC to function only as marginal regulators. With vegetation recovery, increased plant diversity and biomass intensify nitrogen demand and uptake [80], while the pools of mineral nitrogen progressively expand in association with higher SOC and TN retention [41]. Simultaneously, forest recovery promotes soil nitrogen accumulation and maintains NH4⁺ as the dominant inorganic nitrogen form under persistent nitrogen limitation, thereby strengthening the regulatory role of nitrogen fractions in later restoration stages [81]. Consequently, TN and NH4 ⁺ emerge as the primary predictors of soil stoichiometric variation, marking a transition from depth‑dependent and carbon‑limited regulation toward complex nutrient regulation dominated by nitrogen availability and transformation processes [12,82].

The persistent vertical stratification of soil C, N, and P concentrations across restoration stages demonstrates that surface soils remain strongly influenced by litter‑derived organic matter inputs, whereas deeper layers are comparatively constrained by stable parent material conditions. However, the greater dispersion of surface soils (0–20 cm) along the primary ordination axes in later restoration stages reveals increasing heterogeneity in near‑surface nutrient cycling processes. Surface nutrient dynamics are more strongly regulated by soil moisture, bulk density, and texture, whereas deeper soils remain primarily controlled by geo‑climatic background conditions [83]. As restoration advances from herbaceous to woody vegetation, increasingly heterogeneous litter deposition and root inputs intensify spatial variability in surface nutrient distribution [84]. At the same time, enhanced soil moisture and physicochemical weathering in topsoil strengthen localized interactions among water availability, mineral dissolution and re-precipitation, thereby increasing heterogeneity in nutrient cycling near the surface [85]. Moreover, plant‑specific litter inputs, root exudates, localized biogeochemical hotspots, and increasing variability in soil moisture and texture during vegetation recovery jointly contribute to the stronger dispersion of surface soils observed in the later restoration stages [76,86,87].

### 4.3. Validation of successional shifts in depth‑dependent stoichiometric controls

The linear mixed‑effects models confirmed that forest type and soil depth interactively shape soil properties and stoichiometry along the restoration gradient. The higher bulk density in P. yunnanensis than in evergreen forest reflects compaction from coniferous litter and root architecture, which may restrict deeper nutrient cycling [88,89]. Soil organic carbon peaked in the mixed forest and declined with depth, consistent with enhanced litter diversity and root inputs in mid‑succession, while deeper layers remained carbon‑limited. Interestingly, total nitrogen showed no type or depth effect, yet inorganic N (NH_4_⁺ and NO_3_−) was highest in the pine forest, likely due to slower nitrification under acidic conditions or lower microbial N demand, indicating decoupled N pools during early restoration [90–92]. In contrast, total phosphorus was higher in pine and mixed forests, whereas available P was surface‑enriched and maximal in pine, suggesting that early stages retain more labile P, but this availability diminishes as restoration proceeds [93,94]. Stoichiometrically, C:N decreased with depth across all types, reflecting higher recalcitrance in subsoils; C:P was highest in the evergreen forest and surface layer, while N:P was elevated only in the evergreen type, implying progressive P limitation in late succession. The significant Forest × Depth interactions for Al and Fe indicate that their distributions were jointly influenced by vegetation type and soil depth. Higher contents of amorphous Al and Fe in the mixed forest may favor the formation of mineral-associated organic matter, thereby contributing to greater carbon stabilization [11,40,95]. Together, these results quantitatively demonstrate that depth effects on stoichiometry are not uniform but are modulated by forest type, with physical stratification dominating in pine stands and increasingly complex biological and geochemical controls emerging in later stages. This supports the view that restoration progressively shifts regulation from vertical heterogeneity toward integrated plant–soil–mineral feedbacks, reinforcing the findings from RDA and random forest analyses.

### 4.4. Variation in key predictors of soil stoichiometric ratios across successional stages

In the Pinus yunnanensis forest, the RF results revealed that available K and available Al were the primary predictors of C:P, suggesting that P availability is largely governed by colloidal surface reactions rather than vertical heterogeneity—exchangeable Al^3^ ⁺ competes with P for sorption sites on Fe/Al oxides, while K⁺ serves as an indicator of cation exchange capacity [11,96,97]. The control of bulk density and available P over C:N further implies that physical compaction restricts organic matter decomposition and nutrient turnover, whereas the dominance of total nitrogen over N:P indicates that the absolute N pool, rather than depth-related P stability, dictates the N:P balance in these early-stage plantations [15]. As succession proceeds toward coniferous–broadleaved mixed forests, the diversification of significant predictors (including TP and NO_3_− -N) corresponds with increasing litter complexity and root‑mediated nutrient redistribution, which intensifies biotic regulation through litter quality and nutrient cycling and progressively reduces the constraints effect observed in monospecific stands [98]. The additional influence of soil NO_3_− -N on C:P further demonstrates increasing coupling between nitrogen mineralization dynamics and phosphorus availability during succession, consistent with stronger N–P interactions along the restoration trajectory.

The strong predictive role of SOC for both C:P and C:N ratios in mixed forests demonstrates the dominant contribution of organic matter accumulation to stoichiometric regulation during secondary succession, as SOC and TN concentrations increase substantially with restoration while TP remains relatively constrained by parent material weathering and vegetation uptake. Under these conditions, increasing SOC amplifies shifts in both C:P and C:N ratios [11,99,100]. In contrast, the significant effects of nitrogen forms, particularly TN and NO_3_− -N, on N:P variation demonstrate that nitrogen cycling dynamics exert stronger control over N:P stoichiometry than phosphorus supply during the mixed‑forest stage. This pattern also confirms that N:P ratios are more responsive to fluctuations in labile nitrogen pools than to total phosphorus pools, highlighting enhanced sensitivity of nutrient stoichiometry to nitrogen availability during forest recovery [84,101].

The divergent drivers of C:N among forest types demonstrate stage‑dependent shifts in nutrient cycling constraints during succession.In the mixed conifer–broadleaf forest, the significant control of TOC and TN over C:N reflects that coupled carbon–nitrogen accumulation governs organic matter quality during this transitional stage, while the additional role of total Al in predicting C:P indicates that mineral-phase sorption begins to participate in phosphorus dynamics as broadleaf litter inputs increase [102]. In contrast, the mature evergreen broadleaved forest is characterized by stronger biological and chemical regulation of stoichiometric relationships. The significant roles of TOC, pH, and TN in shaping C:N demonstrate that chemical stabilization of organic matter, combined with carbon and nitrogen accumulation, progressively supersedes hydrological regulation during advanced recovery [103]. However, the RF results for this mature stage reveal that C:P is co-regulated by total P, available Al, TOC, and nitrate nitrogen, while N:P is predominantly governed by both total P and total N, suggesting that absolute nutrient reservoir sizes and organo-mineral complexation constitute the primary regulatory pathways in advanced successional ecosystems [104]. Overall, the transition from predominantly abiotic controls in early succession to stronger biological and chemical regulation in later successional stages demonstrates progressively intensified nutrient coordination and internal biogeochemical feedback during forest restoration, with phosphorus‑related constraints becoming increasingly important as ecosystem recovery advances [78,105].

We acknowledge that soil sampling was confined to the single peak growing season (June 2025) and thus did not capture seasonal variations in nutrient availability. Nevertheless, sampling during this biologically active period provides a consistent baseline for comparing restoration-driven changes across forest types, and our interpretations are strictly confined to static chemical associations and physico-chemical equilibria, as microbial biomass and enzymatic activities were not measured. Future multi-seasonal monitoring, ideally combined with microbiological and isotopic approaches, is needed to fully disentangle the temporal and biotic dimensions of stoichiometric dynamics.

## 5. Conclusions

Forest restoration on the southwestern China plateau significantly altered soil C:N:P stoichiometric characteristics and their regulatory patterns across successional stages. SOC/TOC and total nitrogen were the dominant factors controlling soil stoichiometry across all forest types, whereas the effects of Fe/Al oxides, pH, and nutrient fractions varied with vegetation restoration stage. In the Pinus yunnanensis forest, available K and available Al were the primary predictors of C:P, and total nitrogen was the primary predictor of N:P. In the mixed conifer–broadleaf forest, TOC became the primary predictor of C:P and C:N ratios, while nitrogen fractions strongly regulated N:P variation. In the evergreen broadleaf forest, C:P was co-regulated by total P, available Al, TOC, and nitrate nitrogen, while N:P was predominantly governed by total P and total N. Redundancy and random forest analyses showed that restoration increased the explanatory power of environmental variables and strengthened plant-soil nutrient associations along the successional gradient. The significant forest type–depth interactions for Al, Fe, and P stoichiometry further verified that depth effects are modulated by restoration stage, corroborating the shift from physical to biogeochemical controls. Surface soils maintained clear nutrient enrichment across all restoration stages, while nutrient heterogeneity in the 0–20 cm layer increased during later restoration stages. Overall, forest restoration promoted a transition from available K/Al and bulk density constraints in the early-stage plantation toward absolute nutrient pools and organo-mineral interactions in the mature forest.

## Supporting information

S1 Table Stand structural parameters (mean height and diameter at breast height, DBH) of the three forest types (Pinus yunnanenssis forest, mixed conifer–broadleaf forest, and evergreen broadleaf forest) across the nine sampling plots. (XLSX)

S2 Table Variance inflation factor (VIF) values for the environmental predictor variables included in the redundancy analysis (RDA) for each forest type (Pinus yunnanensis, mixed conifer–broadleaf, and evergreen broadleaf). (XLSX)

S1 Fig Normal Q–Q plots for the residuals of the linear mixed‑effects models used to test the effects of forest type, soil depth, and their interaction on soil properties and stoichiometric ratios. (TIF)

S1 Data Data code. (ZIP)
