# Supplementary material for: Successional Shifts in Soil C:N:P stoichiometry and its environmental controls during forest restoration in Huize County, Qujing, Yunnan Province
Source: PLoS One. 2026 Aug 21;21(8):e0356261. doi: 10.1371/journal.pone.0356261 (PMC13496410; doi:10.1371/journal.pone.0356261)
Supplement: Supplementary file 1 [file pone.0356261.s006.docx]

**Original Manuscript Number:** PONE-D-26-28435

**Original Article Title: “**Successional Shifts in Soil C:N:P Stoichiometry and Its Environmental Controls During Forest Restoration in Huize County, Qujing, Yunnan Province”

Dear Editor,

We would like to express our sincere gratitude to you and the reviewers for the valuable time and constructive comments devoted to our manuscript (Manuscript Number: PONE-D-26-28435). The insightful suggestions have been instrumental in helping us significantly improve the quality of our work.

We have carefully considered all the comments and have revised the manuscript accordingly. Our point-by-point responses to each comment are detailed below.

We believe the manuscript is now strengthened and hope it meets the journal's standards for publication.

Thank you for your consideration.

Best regards,

Bo Hu

**Academic Editor, Concern # 1: Please ensure that your manuscript meets PLOS ONE's style requirements, including those for file naming.**

**Author response:** We have carefully followed PLOS ONE’s style templates and ensured that all submitted files—including file names—meet the journal’s requirements. Please let us know if any further adjustments are needed.

**Academic Editor, Concern # 2: Please note that PLOS One has specific guidelines on code sharing for submissions in which author-generated code underpins the findings in the manuscript. In these cases, we expect all author-generated code to be made available without restrictions upon publication of the work. Please review our guidelines and ensure that your code is shared in a way that follows best practice and facilitates reproducibility and reuse.**

**Author response:** We have read the code sharing guidelines carefully and have included all data and author-generated code as supplementary files submitted together with the manuscript. Please let us know if any further revisions are needed.

**Academic Editor, Concern # 3: In your Methods section, please provide additional information regarding the permits you obtained for the work. Please ensure you have included the full name of the authority that approved the field site access and, if no permits were required, a brief statement explaining why.**

**Author response:** We thank the editor for raising this point. In the revised manuscript, we have added a clear statement in the Methods section to explain that no specific permits were required for our field work, as the study sites are openly accessible and involve only non‑destructive sampling.

**Author action:** At the end of Section 2.1 (Profile of the Study Area), we have inserted the following sentence : The study sites are located in collectively owned general forestlands with no protected area designation. They are openly accessible without administrative restrictions, and sampling involved only non‑destructive collection of soil and leaves.

**Academic Editor, Concern # 4: When completing the data availability statement of the submission form, you indicated that you will make your data available on acceptance. We strongly recommend all authors decide on a data sharing plan before acceptance, as the process can be lengthy and hold up publication timelines. Please note that, though access restrictions are acceptable now, your entire data will need to be made freely accessible if your manuscript is accepted for publication. This policy applies to all data except where public deposition would breach compliance with the protocol approved by your research ethics board. If you are unable to adhere to our open data policy, please kindly revise your statement to explain your reasoning and we will seek the editor's input on an exemption. Please be assured that, once you have provided your new statement, the assessment of your exemption will not hold up the peer review process.**

**Author response:** We have discussed the data sharing plan among all co-authors and fully agree to make all data and code available upon publication, in line with PLOS ONE’s policy. We will revise the Data Availability Statement accordingly and do not request any exemption. Please let us know if further changes are needed.

**Academic Editor, Concern # 5: PLOS requires an ORCID iD for the corresponding author in Editorial Manager on papers submitted after December 6th, 2016. Please ensure that you have an ORCID iD and that it is validated in Editorial Manager.**

**Author response:** We confirm that all authors have valid ORCID iDs and that they have been successfully validated in Editorial Manager.

**Academic Editor, Concern # 6: Please ensure that you refer to Figures 1 - 4 in your text as, if accepted, production will need this reference to link the reader to the figure.**

**Author response:** We have reviewed the manuscript and added explicit in‑text citations for Figures 1–4 in the appropriate places to ensure proper linking during production. Please let us know if further changes are required.

**Academic Editor, Concern # 7: We note that Figure 1 in your submission contain map images which may be copyrighted. All PLOS content is published under the Creative Commons Attribution License (CC BY 4.0), which means that the manuscript, images, and Supporting Information files will be freely available online, and any third party is permitted to access, download, copy, distribute, and use these materials in any way, even commercially, with proper attribution. For these reasons, we cannot publish previously copyrighted maps or satellite images created using proprietary data, such as Google software (Google Maps, Street View, and Earth).**

**Author response:** We appreciate the editor’s concern regarding the copyright of Figure 1. We confirm that the base map for Figure 1 was obtained from the Geospatial Data Cloud ([http://www.gscloud.cn](http://www.gscloud.cn/)), operated by the Computer Network Information Center, Chinese Academy of Sciences, and is not derived from any proprietary or copyrighted sources such as Google Maps or other commercial software. We have also attached a screenshot of the data usage and copyright statement from the website as supporting evidence for your reference. We trust this addresses the copyright concern. Please let us know if further modifications are required.

**Author action:** In the revised manuscript, we have already added a clear data source citation in the figure caption: “The data set is provided by Geospatial Data Cloud site, Computer Network Information Center, Chinese Academy of Sciences (http://www.gscloud.cn).”

**Academic Editor, Concern # 8: If the reviewer comments include a recommendation to cite specific previously published works, please review and evaluate these publications to determine whether they are relevant and should be cited. There is no requirement to cite these works unless the editor has indicated otherwise.**

**Author response:** We sincerely thank the editor for this suggestion. We have carefully reviewed the five papers recommended by Reviewer 1. One of them (Li et al., 2025) is directly relevant to our study and has been cited in the revised manuscript. The remaining four papers, while not directly overlapping with our specific focus, were nonetheless carefully read and offered useful conceptual perspectives. Please let us know if any further modifications are required.

**Reviewer#1, Concern # 1: The study includes only three replicate regions per forest type, and soil samples from five points within each plot were composited into a single sample. Please clarify whether the true sample size for statistical analyses was n = 3 or n = 27. The potential issue of pseudoreplication should be discussed because it may inflate statistical significance.**

**Author response:** We sincerely thank the reviewer for raising this important point. We honestly acknowledge that this was a writing error in our initial manuscript. In the actual field design, we established nine independent 20 m × 20 m plots per forest type (three on the upper slope, three on the middle slope, and three on the lower slope), and all statistical analyses—including RDA, Random Forest, and Mantel tests—were performed based on the true data from these nine plots (i.e., n=9 per forest type). The five-point composite sampling was employed solely as a technical procedure to reduce within-plot micro-variability and was never treated as statistical replication. In the revised manuscript, we have corrected the misleading description from "three regions" to "nine plots" in the Methods section, and explicitly defined the plot as the true independent unit for statistical testing in both the Methods and results sections. Therefore, our statistical inferences are firmly based on genuine independent plots, and the concern about pseudoreplication inflating statistical significance does not apply to our study. We apologize for any confusion caused by this descriptive error.

**Author action:**We have corrected the replicate description in Section 2.2.1 by replacing the entire erroneous paragraph with a revised version that changes "three regions" to "nine plots" and updates the sample numbers from 27 to 81 composite samples and from 81 to 243 undisturbed cores. A clarifying statement has also been added within this section to emphasize that the plot (n=9 per forest type) is the true independent unit for statistical analyses. Additionally, in Section 3.2, we have supplemented the beginning of the RDA results with a sentence specifying that the analysis was based on plot-level replicates. All corresponding changes have been highlighted in the revised manuscript.

**Reviewer#2, Concern # 2: The study interprets differences among forest types as restoration-stage effects. However, the manuscript does not provide evidence that the three forest types represent a true successional sequence with similar historical land-use conditions. More justification is needed to support this assumption.**

**Author response:** We sincerely thank the reviewer for this critical and constructive comment. We fully agree that the assumption of a true successional sequence with comparable historical and environmental backgrounds must be rigorously justified when employing a space-for-time substitution approach. In response, we have undertaken three complementary modifications to strengthen this rationale. First, in the Introduction (Section 1), we added literature citations in the Introduction to establish the regional representativeness of this successional trajectory. Second, in Materials and Methods (Section 2.2.1), we have added quantitative stand structural parameters—specifically, mean tree height and diameter at breast height (DBH)—which exhibit clear increasing gradients across the three forest types, providing direct empirical evidence that these communities represent structurally distinct and progressively more advanced restoration stages. Third, in the Discussion (Section 4), we have included a cautious acknowledgment of the inherent limitations of the space-for-time substitution approach, while also clarifying that our plot selection minimized variations in topography, soil parent material, and climatic background, thereby supporting the inference that observed differences primarily reflect restoration progression rather than inherent site heterogeneity.

**Author action:** 1. In the Introduction (Section 1), we added the following text: In the study region, previous phytosociological studies have demonstrated that secondary succession following anthropogenic disturbance typically follows a predictable trajectory from pioneer Pinus yunnanensis forests, through mixed conifer–broadleaf transitional communities, towards the zonal semi-humid evergreen broadleaved forests[46]. This well-documented successional framework provides a robust and ideal chronosequence for examining how plant–soil nutrient associations and soil stoichiometric regulation evolve during ecosystem recovery. Moreover, the successional classification of tree species utilized in local restoration trials further corroborates the functional differentiation of these seral stages, reinforcing their suitability for such analysis[47].

2.In Materials and Methods (Section 2.2.1), we inserted the following text after describing the plot establishment, together with a new Supplementary Table S1 containing detailed stand structural parameters: In addition, the three forest types exhibited clear structural gradients consistent with successional progression: the mean stand height and diameter at breast height (DBH) increased progressively from the Pinus yunnanensis forest (height: 10.9 ± 0.9 m; DBH: 87.3 ± 2.8 mm) through the mixed conifer–broadleaf forest (height: 11.1 ± 0.9 m; DBH: 92.5 ± 7.6 mm) to the evergreen broadleaf forest (height: 11.6 ± 0.6 m; DBH: 96.1 ± 10.8 mm), further supporting their designation as distinct restoration stages (detailed stand structural parameters are provided in Table S1).

3.In the Discussion (Section 4), we added the following paragraph at the end to acknowledge methodological limitations while reinforcing the validity of our approach: We acknowledge that soil sampling was confined to the single peak growing season (June 2025) and thus did not capture seasonal variations in nutrient availability. Nevertheless, sampling during this biologically active period provides a consistent baseline for comparing restoration-driven changes across forest types, and our interpretations are strictly confined to static chemical associations and physico-chemical equilibria, as microbial biomass and enzymatic activities were not measured. Future multi-seasonal monitoring, ideally combined with microbiological and isotopic approaches, is needed to fully disentangle the temporal and biotic dimensions of stoichiometric dynamics.

**Reviewer#1, Concern # 3: Soil samples were collected only in June 2025, while plant samples were collected in June and November 2025. Seasonal and interannual variability can strongly affect nutrient availability and stoichiometry. The limitation of single-year sampling should be clearly acknowledged.**

**Author response:** We sincerely thank the reviewer for this valuable comment. We fully agree that seasonal and interannual variability can affect soil nutrient availability, and we acknowledge this limitation explicitly. Accordingly, we have added a concise paragraph at the end of the Discussion section to address this issue, while also providing a brief justification for our sampling strategy during the peak growing season.

**Author action:** We added one short paragraph at the end of the Discussion section to acknowledge that soil sampling was confined to June 2025 and did not capture seasonal variations, while noting that the peak growing season provides a consistent baseline for comparing restoration stages and calling for future multi-seasonal monitoring.

**Added text:** We acknowledge that soil sampling was confined to the single peak growing season (June 2025) and thus did not capture seasonal variations in nutrient availability. Nevertheless, sampling during this biologically active period provides a consistent baseline for comparing restoration-driven changes across forest types, and our interpretations are strictly confined to static chemical associations and physico-chemical equilibria, as microbial biomass and enzymatic activities were not measured. Future multi-seasonal monitoring, ideally combined with microbiological and isotopic approaches, is needed to fully disentangle the temporal and biotic dimensions of stoichiometric dynamics.

**Reviewer#1, Concern # 4: The RDA results report explained variances of 99.11% and 99.62% for the mixed forest and evergreen broadleaf forest, respectively. Such extremely high explanatory power is unusual in ecological field studies and may indicate overfitting or multicollinearity among explanatory variables. The authors should verify these results and provide variance inflation factor (VIF) analysis.**

**Author response:** We sincerely thank the reviewer for this rigorous and highly constructive statistical comment.To rigorously address the potential overfitting and multicollinearity issues, we implemented a strict Variance Inflation Factor (VIF) screening procedure. Explanatory variables with VIF > 10 were sequentially removed from the models to eliminate redundant collinearity(the detailed VIF values and screening results are now provided in Table S1 of the Supplementary Materials).

**Reviewer#1, Concern # 5: The Random Forest model uses soil C, C, and N ratios as predictor variables when explaining other stoichiometric ratios. This may introduce circular reasoning because response variables are mathematically related to predictor variables. The modeling strategy should be reconsidered and justified.**

**Author response:** We sincerely thank the reviewer for identifying this critical statistical issue. We fully agree that using mathematically interrelated ratio variables (e.g., using C:N and N:P to predict C:P) introduces circularity and may obscure true ecological mechanisms. Following the reviewer's suggestion, we have thoroughly reconsidered and reconstructed our modeling strategy, re-ran all relevant analyses, and systematically revised the corresponding texts in the Results, Discussion, Conclusion, and Abstract sections. The revised results now provide a more robust and ecologically meaningful interpretation of the regulatory mechanisms governing soil C:N:P stoichiometry across the restoration gradient.

**Author action:**1. We removed all mathematically derived ratio variables (i.e., soil C:N, C:P, and N:P) from the predictor matrix of the Random Forest and RDA models, retaining only independently measured raw variables (e.g., [S.TN](https://s.tn/), [S.TP](https://s.tp/), S.AK, S.AAl, BD, pH, TOC, and nitrogen fractions) to ensure that all explanatory powers reflect genuine environmental and biological controls rather than mathematical artifacts.

2. We re-ran the Random Forest and RDA analyses using the revised variable set. The updated results revealed substantially different regulatory patterns: in the Pinus yunnanensis forest, available K and available Al (rather than soil layer) emerged as the primary predictors of C:P, and total nitrogen became the primary predictor of N:P; in the evergreen broadleaf forest, total P and total N (rather than plant N:P or soil C:P) predominantly governed N:P, while C:P was co-regulated by total P, available Al, TOC, and nitrate nitrogen.
3. Based on these new results, we completely rewrote Section 3.3 (Key Predictors of Soil Stoichiometric Ratios Across Restoration Stages) and substantially revised Section 4.3 (Variation in Key Predictors of Soil Stoichiometric Ratios Across Successional Stages) to reflect the non-circular findings, with all interpretations now grounded in mechanistic soil chemistry and nutrient stock limitations.
4. We accordingly updated the Conclusion and Abstract to ensure consistency with the revised results, replacing the previous depth-dependent narrative for the P. yunnanensis forest with the revised K/Al and TN constraints, and revising the evergreen broadleaf forest description from plant-mediated feedbacks to absolute nutrient pool and organo-mineral interaction controls.
5. All related figures have been updated accordingly, and the revised manuscript now presents a statistically sound and ecologically coherent interpretation of the data.

**Reviewer#1, Concern # 6: Only leaf N, P, C, and N ratios were measured. However, the manuscript repeatedly discusses plant–soil nutrient coupling. Additional plant variables such as biomass, litter quality, or nutrient stocks would strengthen these conclusions.**

**Author response:** We fully agree with the reviewer’s assessment. Our original use of the term “plant–soil nutrient coupling” was indeed overly strong, given that our dataset includes leaf nutrient concentrations but not biomass, litter quality, or nutrient stocks.

**Author action:** To strictly align our conclusions with the empirical data, we have systematically revised the terminology throughout the manuscript (Abstract, Introduction, Discussion, and Conclusions). Specifically, we have replaced “coupling” with more data-appropriate terms such as “plant–soil nutrient associations” and “leaf–soil stoichiometric coordination”, and we have rephrased causal inferences into descriptive statements. These revisions ensure that our discussion of plant–soil relationships remains within the correlative framework supported by our redundancy and random forest analyses.

**Reviewer#1, Concern # 7: Soil depth was identified as a major driver of stoichiometric variation, yet no separate analysis was performed to compare depth effects among forest types. A mixed-effects model or two-way ANOVA considering forest type and soil depth interactions would provide stronger evidence.**

**Author response:** We sincerely thank the reviewer for this valuable suggestion. We fully agree that explicitly testing the interactive effects of forest type and soil depth is essential to provide stronger statistical support for our conclusion that depth-dependent stoichiometric regulation shifts along the restoration gradient. Following this constructive recommendation, we performed linear mixed‑effects models and the results indeed revealed significant forest type × depth interactions for several key variables, particularly Al, Fe, and P‑related stoichiometric ratios, confirming that depth effects on nutrient balance are modulated by restoration stage. These findings not only corroborate our original conclusions but also provide a more rigorous statistical foundation for the successional shift from physical stratification‑dominated controls in early pine forests toward more integrated biogeochemical regulations in mature stands.

**Author action:** 1. We conducted linear mixed‑effects models using the lme4 package in R, with forest type, soil depth, and their interaction as fixed effects, to formally test the interactive effects on soil properties and stoichiometric ratios.

2. We added a new subsection (3.3) in the Results section to present the mixed‑model outputs, including the significant Forest × Layer interactions for Al, Fe, and P‑related stoichiometric ratios, accompanied by two new figures: Fig. 4 interactive effects of forest type and soil depth on selected soil properties; Fig. 5 Main effects of forest type on selected soil properties across three depth layers (1: 0–20, 2: 20–50, 3: 50–80 cm).
3. We added a corresponding new subsection (4.3) in the Discussion section to interpret the ecological implications of these interactions, supporting the successional shift from physical to biogeochemical controls.
4. We supplemented the Abstract, Materials and Methods, and Conclusions with brief statements about the mixed‑model validation, along with updated references and renumbering of all subsections and citations accordingly.

**Reviewer#1, Concern # 8: Several references are extremely recent (2025–2026), and some appear unusual or difficult to verify. The authors should carefully confirm the accuracy, publication status, and relevance of all references before publication. It is suggested to cite recent ciations such as**

**“https://doi.org/10.1016/j.catena.2023.107357;**

**https://doi.org/10.1038/s41467-025-65773-1;**

**https://doi.org/10.1007/s42773-025-00540-5;**

**https://doi.org/10.1016/j.cj.2026.01.002;**

**https://doi.org/10.1016/j.eiar.2026.108503”**

**Author response:** We sincerely thank the reviewer for this valuable suggestion. We have carefully read and evaluated each of the recommended papers. Among them, we found that the study by Li et al. (2025) on silicate weathering-derived phosphorus release and its mitigation of global phosphorus limitation is highly relevant to our discussion of soil C:N:P stoichiometric regulation and its ecological implications in the context of forest restoration. We have therefore incorporated this reference into the Introduction section to strengthen the broader context of phosphorus dynamics. Regarding the other four recommended papers—Liu et al. (2023, Catena) on soil C:P ratio regulating stream water nitrogen and phosphorus, Yuan et al. (2026, Biochar) on phosphorus-modified biochar for heavy metal remediation, Cheng et al. (2025, The Crop Journal) on synthetic biology approaches for nitrogen and carbon fixation, and Zhi et al. (2026, Environmental Impact Assessment Review) on nitrogen metabolism in coastal wetland socio-ecological systems—while they do not directly overlap with our specific research focus on forest restoration succession in plateau ecosystems, we have nonetheless studied them carefully and benefited from their conceptual insights. We appreciate the reviewer's thoughtful suggestions.

**Author action:** We have re-verified all 2025–2026 references originally cited in our manuscript, confirmed their publication status, and supplemented complete volume numbers, page ranges, and DOI information where necessary to ensure full verifiability. Moreover, we have added the reference Li et al. (2025) to the Introduction section.

**Added text:** Recent global-scale evidence further indicates that phosphorus release from silicate rock weathering can mitigate phosphorus limitation of vegetation growth, with a relative contribution of approximately 15.5%[24].

**Reviewer#1, Concern # 9: The quality of the figures are very low, please imrpove the figures quality.**

**Author response:** We sincerely thank the reviewer for pointing out the blurry figures in the PDF proof.We would like to clarify that this blurriness is caused by the journal's online submission system, which automatically compresses the high-resolution TIFF files during the PDF generation process for review purposes. Our original figures were exported as high-resolution TIFF , and they are perfectly clear in their native format.

**Author action:** To demonstrate the true quality, we have attached the original high-resolution figures (Figures 1–4) directly within this response letter (please see below). Additionally, to proactively mitigate the system's compression, we have re-exported all TIFF files with an even higher resolution to balance file size, ensuring the final published version will meet the journal's high standards. We hope the reviewer can assess the actual quality via the attached images in this response.

**Fig 1. Location of the study area in Huize County, Qujing City, Yunnan Province, China.**The data set is provided by Geospatial Data Cloud site, Computer Network Information Center, Chinese Academy of Sciences(http://www.gscloud.cn).

**Fig 2. Overall Mantel and Pearson correlation analysis of soil properties and soil C:N:P stoichiometric ratios in Pinus yunnanensis forest(a), mixed conifer–broadleaf forest(b), and evergreen broadleaf forest(c).**

**Fig 3. Redundancy analysis (RDA) of soil C:N:P stoichiometric ratios and environmental variables in Pinus yunnanensis forest(a), mixed conifer–broadleaf forest(b), and evergreen broadleaf forest(c).**

**Fig 4. Interactive effects of forest type and soil depth on selected soil properties and stoichiometric ratios.**

**Fig 5. Main effects of forest type on selected soil properties across three depth layers (1: 0–20, 2: 20–50, 3: 50–80 cm).**

**Fig 6. Relative importance of environmental variables in predicting soil C:N:P stoichiometric ratios in Pinus yunnanensis forest(a), mixed conifer–broadleaf forest(b), and evergreen broadleaf forest(c) based on Random Forest analysis.**

**Reviewer#2, Concern # 1: In the introduction, previous studies have shown that litter has an impact on nitrogen content and nitrogen cycling efficiency. The study only focuses on leaf nutrients and cannot fully represent the coupling of plant-soil nutrients. It is recommended to add indicators such as litter and improve relevant data.**

**Author response:** We fully agree with the reviewer that our introduction contained several statements that overemphasized the causal role of litter inputs in regulating nitrogen dynamics (e.g., “thereby reshaping nitrogen dynamics,” “because of enhanced litter inputs”), whereas our dataset does not include direct litter measurements.

**Author action:** To eliminate this mismatch, we have carefully revised the Introduction by rephrasing these causal assertions into literature-based background statements. Specifically, we have replaced phrases such as “thereby reshaping” with “which are known to influence,” and “because of” with “a pattern often attributed to” or “which has been associated with.” We have also modified the sentence linking “low-quality litter inputs” to “enhanced nitrogen cycling” by changing the causal parallel into a descriptive association. These revisions ensure that the Introduction accurately sets the context for our study without implying that we directly tested litter-mediated mechanisms, while still properly acknowledging the established ecological roles of litter from previous research.

**Reviewer#2, Concern # 2: It is recommended to systematically review the existing research gaps and precisely highlight the unique innovations of this study.**

**Author response:** We thank the reviewer for this constructive suggestion. We agree that the original manuscript did not adequately articulate the research gaps or clearly highlight the innovations of this study. In the revised version, we have addressed these issues accordingly.

**Author action:** We have restructured the latter part of the Introduction by adding a structured summary that explicitly identifies three major limitations in previous studies: (1) lack of continuous successional gradient analysis; (2) insufficient quantification of the shifting roles of Fe/Al oxides and pH across stages; and (3) limited direct multivariate evidence for the transition of regulatory mechanisms. Correspondingly, we have added a concise paragraph at the end of the introduction stating the two main innovations of this study, including the systematic assessment along a complete successional gradient and the integrated multivariate approach that quantifies the shifting importance of multiple environmental factors.

**Added text:** The innovations of this study are twofold. First, it systematically assesses soil stoichiometric regulation along a complete successional gradient under uniform environmental background, a context rarely examined in this region. Second, by integrating Mantel tests, RDA, and random forest, it quantifies the shifting importance of depth, carbon, nitrogen, Fe/Al oxides, and pH, revealing a transition from depth-dominated to nitrogen- and pH-mediated controls.

**Reviewer#2, Concern # 3: This study focuses on the chemometric succession patterns of soil carbon and nitrogen during forest restoration. However, the article only uses "Yunnan pine forest → mixed coniferous and broad-leaved forest → evergreen broad-leaved forest" to represent the restoration time gradient, without comparing the chronological gradient of plots, which presents limitations.**

**Author response:** We sincerely thank the reviewer for this critical and constructive comment. We fully agree that the assumption of a true successional sequence with comparable historical and environmental backgrounds must be rigorously justified when employing a space-for-time substitution approach. In response, we have undertaken three complementary modifications to strengthen this rationale. First, in the Introduction (Section 1), we added literature citations in the Introduction to establish the regional representativeness of this successional trajectory. Second, in Materials and Methods (Section 2.2.1), we have added quantitative stand structural parameters—specifically, mean tree height and diameter at breast height (DBH)—which exhibit clear increasing gradients across the three forest types, providing direct empirical evidence that these communities represent structurally distinct and progressively more advanced restoration stages. Third, in the Discussion (Section 4), we have included a cautious acknowledgment of the inherent limitations of the space-for-time substitution approach, while also clarifying that our plot selection minimized variations in topography, soil parent material, and climatic background, thereby supporting the inference that observed differences primarily reflect restoration progression rather than inherent site heterogeneity.

**Author action:** 1. In the Introduction (Section 1), we added the following text: In the study region, previous phytosociological studies have demonstrated that secondary succession following anthropogenic disturbance typically follows a predictable trajectory from pioneer Pinus yunnanensis forests, through mixed conifer–broadleaf transitional communities, towards the zonal semi-humid evergreen broadleaved forests[46]. This well-documented successional framework provides a robust and ideal chronosequence for examining how plant–soil nutrient associations and soil stoichiometric regulation evolve during ecosystem recovery. Moreover, the successional classification of tree species utilized in local restoration trials further corroborates the functional differentiation of these seral stages, reinforcing their suitability for such analysis[47].

2. In Materials and Methods (Section 2.2.1), we inserted the following text after describing the plot establishment, together with a new Supplementary Table S1 containing detailed stand structural parameters: In addition, the three forest types exhibited clear structural gradients consistent with successional progression: the mean stand height and diameter at breast height (DBH) increased progressively from the Pinus yunnanensis forest (height: 10.9 ± 0.9 m; DBH: 87.3 ± 2.8 mm) through the mixed conifer–broadleaf forest (height: 11.1 ± 0.9 m; DBH: 92.5 ± 7.6 mm) to the evergreen broadleaf forest (height: 11.6 ± 0.6 m; DBH: 96.1 ± 10.8 mm), further supporting their designation as distinct restoration stages (detailed stand structural parameters are provided in Table S1).
3. In the Discussion (Section 4), we added the following paragraph at the end to acknowledge methodological limitations while reinforcing the validity of our approach: We acknowledge that soil sampling was confined to the single peak growing season (June 2025) and thus did not capture seasonal variations in nutrient availability. Nevertheless, sampling during this biologically active period provides a consistent baseline for comparing restoration-driven changes across forest types, and our interpretations are strictly confined to static chemical associations and physico-chemical equilibria, as microbial biomass and enzymatic activities were not measured. Future multi-seasonal monitoring, ideally combined with microbiological and isotopic approaches, is needed to fully disentangle the temporal and biotic dimensions of stoichiometric dynamics.

**Reviewer#2, Concern # 4: For the discussion section, delete discussions on relevant data that were not studied in this research. For example, this article does not include data on microorganisms or enzymes, yet the discussion section cites discussions on related mechanisms such as microbial decomposition. It is recommended to add comparative discussions with other areas in the discussion section to clarify the differentiated conclusions of this research.**

**Author response:** We sincerely thank the reviewer for this perceptive and critical comment. We fully concur that invoking microbial and enzymatic mechanisms without supporting experimental evidence is methodologically inappropriate. Guided by this valuable suggestion, we have fundamentally restructured the interpretive logic of our Discussion section through three coordinated revisions. First, we systematically eliminated all speculations pertaining to unmeasured variables (microbial biomass, enzyme activities, and gross mineralization/nitrification rates). Second, we re-anchored our interpretations strictly on measurable physicochemical proxies, focusing exclusively on Fe/Al oxide chemistry, pH equilibria, bulk density, and nutrient pool associations—confining our discussion to static stoichiometric correlations and physico-chemical equilibria rather than dynamic process inferences. Third, we inserted a dedicated comparative paragraph to articulate the differentiated conclusion of our study, highlighting the unique mineral-surface ligand exchange mechanism operating in our acidic, Fe/Al-rich system in contrast to alkaline/calcareous soil systems. In addition, we proactively added a sentence in the Limitations section to explicitly delimit the scope of our inferences.

**Author action:** We have systematically removed all microbiological and enzymatic references throughout the Discussion section. Specifically, all mentions of "microbial activity," "extracellular enzyme activities," "microbial C/P co-limitation," and "mineralization rates" have been deleted and replaced with appropriate physicochemical descriptors. We have also inserted an independent comparative paragraph at the end of subsection 4.1, which explicitly contrasts the regulatory pathway of our acidic system (Fe/Al sorption) with that of alkaline/calcareous systems (carbonate buffering), and clearly identifies mineral-surface sorption processes as our core differentiated conclusion. Additionally, we have added one sentence to the Limitations section, explicitly acknowledging that microbial biomass and enzyme activities were not measured, thereby strictly circumscribing our discussion within the boundaries of static chemical equilibria.

**Added text:** Mechanistically, in contrast to alkaline or calcareous soil systems where stoichiometric ratios are primarily modulated by carbonate buffering and calcium-mediated organic matter stabilization, our acidic, Fe/Al-rich forest soils exhibit a fundamentally different regulatory pathway. Here, the surface sorption and desorption reactions of Fe/Al (hydr)oxides directly govern phosphorus availability and carbon retention, rather than base cation weathering or moisture-limited decomposition. This mineral-surface ligand exchange mechanism constitutes the key differentiated conclusion emerging from our physicochemical data, implying that in humid subtropical acidic forests, the management of organo-mineral complexes may be more critical for nutrient regulation than solely adjusting bulk pH or total nutrient inputs.

**Reference**

[1] Li, C.; Lu, X.; Chen, J.; Tan, Q.; Luo, G.; Wu, L.; Chen, F.; Ran, C.; Zhang, S.; Xiong, L.; Du, C.; Song, F.; Xiao, B.; Li, Z.; Xue, Y.; Long, M.; Bai, X. Silicate Chemical Weathering Disrupts the Global Patterns of Phosphorus Limitation. Nat. Commun. 2025, 16 (1), 10742. https://doi.org/10.1038/s41467-025-65773-1.

[2] Liu, J.; Wang, Y.; Li, Y.; Peñuelas, J.; Zhao, Y.; Sardans, J.; Tetzlaff, D.; Liu, J.; Liu, X.; Yuan, H.; Li, Y.; Chen, J.; Wu, J. Soil Ecological Stoichiometry Synchronously Regulates Stream Nitrogen and Phosphorus Concentrations and Ratios. CATENA 2023, 231, 107357. https://doi.org/10.1016/j.catena.2023.107357.

[3] Yuan, J.; Liu, Y.; He, Q.; Wen, H.; Li, Z.; Lin, R.; Chu, T.; Peng, C.; Zheng, C.; Chen, H.; Tan, Y. Phosphorus-Modified Biochar from Salvia Miltiorrhiza Dregs: Synthesis, Characterization, and Dual-Functional Synergy for Heavy Metal Immobilization and Soil Fertility Augmentation. Biochar 2026, 8 (1), 30. https://doi.org/10.1007/s42773-025-00540-5.

[4] Cheng, Q.; Ma, C.; Wang, X. Biological Nitrogen and Carbon Fixation: Bridging the Gap between Synthetic Symbioses and Synthetic Biology. Crop J. 2026, 14 (1), 74–86. https://doi.org/10.1016/j.cj.2026.01.002.

[5] Zhi, L.; Li, X.; Bai, J.; Wang, D.; Yang, J.; Ren, J. Unveiling Critical Nitrogen Metabolism Nodes and Stakeholder Priorities across Coastal Wetland Eco-Socioeconomic Systems via Integrated Network Analytics. Environ. Impact Assess. Rev. 2026, 121, 108503. https://doi.org/10.1016/j.eiar.2026.108503.
