# Supplementary material for: Successional Shifts in Soil C:N:P stoichiometry and its environmental controls during forest restoration in Huize County, Qujing, Yunnan Province
Source: PLoS One. 2026 Aug 21;21(8):e0356261. doi: 10.1371/journal.pone.0356261 (PMC13496410; doi:10.1371/journal.pone.0356261)
Supplement: Peer review history

# Peer review: Decision Letter 0

**DOI:** 10.1371/journal.pone.0356261.r001

**Authors:** Jian Liu

21 Jun 2026

Dear Dr. Hu,

Thank you for submitting your manuscript to PLOS ONE. After careful consideration, we feel that it has merit but does not fully meet PLOS ONE’s publication criteria as it currently stands. Therefore, we invite you to submit a revised version of the manuscript that addresses the points raised during the review process.

==============================

**ACADEMIC EDITOR:**

==============================

Please submit your revised manuscript by Aug 20 2026 11:59PM. If you will need more time than this to complete your revisions, please reply to this message or contact the journal office at plosone@plos.org. When you're ready to submit your revision, log on to https://www.editorialmanager.com/pone/ and select the 'Submissions Needing Revision' folder to locate your manuscript file.

Please include the following items when submitting your revised manuscript:

- A letter that responds to each point raised by the academic editor and reviewer(s). You should upload this letter as a separate file labeled 'Response to Reviewers'.
- A marked-up copy of your manuscript that highlights changes made to the original version. You should upload this as a separate file labeled 'Revised Manuscript with Track Changes'.
- An unmarked version of your revised paper without tracked changes. You should upload this as a separate file labeled 'Manuscript'.

If you would like to make changes to your financial disclosure, please include your updated statement in your cover letter. Guidelines for resubmitting your figure files are available below the reviewer comments at the end of this letter.

If applicable, we recommend that you deposit your laboratory protocols in protocols.io to enhance the reproducibility of your results. Protocols.io assigns your protocol its own identifier (DOI) so that it can be cited independently in the future. For instructions see: https://journals.plos.org/plosone/s/submission-guidelines#loc-laboratory-protocols. Additionally, PLOS ONE offers an option for publishing peer-reviewed Lab Protocol articles, which describe protocols hosted on protocols.io. Read more information on sharing protocols at https://plos.org/protocols?utm_medium=editorial-email&utm_source=authorletters&utm_campaign=protocols.

As the corresponding author, your ORCID iD is verified in the submission system and will appear in the published article. PLOS supports the use of ORCID, and we encourage all coauthors to register for an ORCID iD and use it as well. Please encourage your coauthors to verify their ORCID iD within the submission system before final acceptance, as unverified ORCID iDs will not appear in the published article. *Only*  the individual author can complete the verification step; PLOS staff *cannot*  verify ORCID iDs on behalf of authors.

We look forward to receiving your revised manuscript.

Kind regards,

Jian Liu

Academic Editor

PLOS One

Journal Requirements:

When submitting your revision, we need you to address these additional requirements.

1. Please ensure that your manuscript meets PLOS ONE's style requirements, including those for file naming. The PLOS ONE style templates can be found at

https://journals.plos.org/plosone/s/file?id=wjVg/PLOSOne_formatting_sample_main_body.pdf and

https://journals.plos.org/plosone/s/file?id=ba62/PLOSOne_formatting_sample_title_authors_affiliations.pdf

2. Please note that PLOS One has specific guidelines on code sharing for submissions in which author-generated code underpins the findings in the manuscript. In these cases, we expect all author-generated code to be made available without restrictions upon publication of the work. Please review our guidelines at https://journals.plos.org/plosone/s/materials-and-software-sharing#loc-sharing-code and ensure that your code is shared in a way that follows best practice and facilitates reproducibility and reuse.

3. In your Methods section, please provide additional information regarding the permits you obtained for the work. Please ensure you have included the full name of the authority that approved the field site access and, if no permits were required, a brief statement explaining why.

4. When completing the data availability statement of the submission form, you indicated that you will make your data available on acceptance. We strongly recommend all authors decide on a data sharing plan before acceptance, as the process can be lengthy and hold up publication timelines. Please note that, though access restrictions are acceptable now, your entire data will need to be made freely accessible if your manuscript is accepted for publication. This policy applies to all data except where public deposition would breach compliance with the protocol approved by your research ethics board. If you are unable to adhere to our open data policy, please kindly revise your statement to explain your reasoning and we will seek the editor's input on an exemption. Please be assured that, once you have provided your new statement, the assessment of your exemption will not hold up the peer review process.

5. PLOS requires an ORCID iD for the corresponding author in Editorial Manager on papers submitted after December 6th, 2016. Please ensure that you have an ORCID iD and that it is validated in Editorial Manager. To do this, go to ‘Update my Information’ (in the upper left-hand corner of the main menu), and click on the Fetch/Validate link next to the ORCID field. This will take you to the ORCID site and allow you to create a new iD or authenticate a pre-existing iD in Editorial Manager.

6. Please ensure that you refer to Figures 1 - 4 in your text as, if accepted, production will need this reference to link the reader to the figure.

7. We note that Figure 1 in your submission contain map images which may be copyrighted. All PLOS content is published under the Creative Commons Attribution License (CC BY 4.0), which means that the manuscript, images, and Supporting Information files will be freely available online, and any third party is permitted to access, download, copy, distribute, and use these materials in any way, even commercially, with proper attribution. For these reasons, we cannot publish previously copyrighted maps or satellite images created using proprietary data, such as Google software (Google Maps, Street View, and Earth). For more information, see our copyright guidelines: http://journals.plos.org/plosone/s/licenses-and-copyright.

We require you to either (1) present written permission from the copyright holder to publish these figures specifically under the CC BY 4.0 license, or (2) remove the figures from your submission:

1. You may seek permission from the original copyright holder of Figure(s) [#] to publish the content specifically under the CC BY 4.0 license.

We recommend that you contact the original copyright holder with the Content Permission Form (http://journals.plos.org/plosone/s/file?id=7c09/content-permission-form.pdf) and the following text:

“I request permission for the open-access journal PLOS ONE to publish XXX under the Creative Commons Attribution License (CCAL) CC BY 4.0 (http://creativecommons.org/licenses/by/4.0/). Please be aware that this license allows unrestricted use and distribution, even commercially, by third parties. Please reply and provide explicit written permission to publish XXX under a CC BY license and complete the attached form.”

Please upload the completed Content Permission Form or other proof of granted permissions as an "Other" file with your submission.

In the figure caption of the copyrighted figure, please include the following text: “Reprinted from [ref] under a CC BY license, with permission from [name of publisher], original copyright [original copyright year].”

2. If you are unable to obtain permission from the original copyright holder to publish these figures under the CC BY 4.0 license or if the copyright holder’s requirements are incompatible with the CC BY 4.0 license, please either i) remove the figure or ii) supply a replacement figure that complies with the CC BY 4.0 license. Please check copyright information on all replacement figures and update the figure caption with source information. If applicable, please specify in the figure caption text when a figure is similar but not identical to the original image and is therefore for illustrative purposes only.

The following resources for replacing copyrighted map figures may be helpful:

USGS National Map Viewer (public domain): http://viewer.nationalmap.gov/viewer/

The Gateway to Astronaut Photography of Earth (public domain): http://eol.jsc.nasa.gov/sseop/clickmap/

Maps at the CIA (public domain): https://www.cia.gov/library/publications/the-world-factbook/index.html and https://www.cia.gov/library/publications/cia-maps-publications/index.html

NASA Earth Observatory (public domain): http://earthobservatory.nasa.gov/

Landsat: http://landsat.visibleearth.nasa.gov/

USGS EROS (Earth Resources Observatory and Science (EROS) Center) (public domain): http://eros.usgs.gov/#

Natural Earth (public domain): http://www.naturalearthdata.com/

8. If the reviewer comments include a recommendation to cite specific previously published works, please review and evaluate these publications to determine whether they are relevant and should be cited. There is no requirement to cite these works unless the editor has indicated otherwise.

[Note: HTML markup is below. Please do not edit.]

Reviewers' comments:

Reviewer's Responses to Questions

**Comments to the Author**

1. Is the manuscript technically sound, and do the data support the conclusions?

Reviewer #1: Yes

Reviewer #2: Partly

**********

2. Has the statistical analysis been performed appropriately and rigorously?

Reviewer #1: Yes

Reviewer #2: Yes

**********

3. Have the authors made all data underlying the findings in their manuscript fully available?

The PLOS Data policy

Reviewer #1: Yes

Reviewer #2: Yes

**********

4. Is the manuscript presented in an intelligible fashion and written in standard English?

Reviewer #1: Yes

Reviewer #2: Yes

**********

Reviewer #1: The article entitled "Soil C:N Stoichiometric Characteristics and Their Environmental Drivers Across Forest Restoration Stages on the Southwestern China Plateau" investigates changes in soil carbon, nitrogen, and phosphorus stoichiometry during forest succession from Pinus yunnanensis forest to mixed conifer–broadleaf forest and evergreen broadleaf forest. The authors aim to identify the environmental factors regulating soil C:N ratios and to understand how nutrient cycling mechanisms shift during forest restoration. The topic is relevant to forest ecology, ecosystem restoration, and nutrient cycling, and it fits well within the scope of the journal. The manuscript contains valuable field data and extensive statistical analyses. However, several methodological, analytical, and interpretational issues should be addressed to improve the scientific rigor and reliability of the study before publication.

1.The study includes only three replicate regions per forest type, and soil samples from five points within each plot were composited into a single sample. Please clarify whether the true sample size for statistical analyses was n = 3 or n = 27. The potential issue of pseudoreplication should be discussed because it may inflate statistical significance.

2.The study interprets differences among forest types as restoration-stage effects. However, the manuscript does not provide evidence that the three forest types represent a true successional sequence with similar historical land-use conditions. More justification is needed to support this assumption.

3.Soil samples were collected only in June 2025, while plant samples were collected in June and November 2025. Seasonal and interannual variability can strongly affect nutrient availability and stoichiometry. The limitation of single-year sampling should be clearly acknowledged.

4.The RDA results report explained variances of 99.11% and 99.62% for the mixed forest and evergreen broadleaf forest, respectively. Such extremely high explanatory power is unusual in ecological field studies and may indicate overfitting or multicollinearity among explanatory variables. The authors should verify these results and provide variance inflation factor (VIF) analysis.

5.The Random Forest model uses soil C, C, and N ratios as predictor variables when explaining other stoichiometric ratios. This may introduce circular reasoning because response variables are mathematically related to predictor variables. The modeling strategy should be reconsidered and justified.

6.Only leaf N, P, C, and N ratios were measured. However, the manuscript repeatedly discusses plant–soil nutrient coupling. Additional plant variables such as biomass, litter quality, or nutrient stocks would strengthen these conclusions.

7.Soil depth was identified as a major driver of stoichiometric variation, yet no separate analysis was performed to compare depth effects among forest types. A mixed-effects model or two-way ANOVA considering forest type and soil depth interactions would provide stronger evidence.

8.Several references are extremely recent (2025–2026), and some appear unusual or difficult to verify. The authors should carefully confirm the accuracy, publication status, and relevance of all references before publication. It is suggested to cite recent ciations such as “https://doi.org/10.1016/j.catena.2023.107357; https://doi.org/10.1038/s41467-025-65773-1; 10.1007/s42773-025-00540-5; https://doi.org/10.1016/j.cj.2026.01.002 ; https://doi.org/10.1016/j.eiar.2026.108503

9. The quality of the figures are very low, please imrpove the figures quality.

Reviewer #2: 1.In the introduction, previous studies have shown that litter has an impact on nitrogen content and nitrogen cycling efficiency. The study only focuses on leaf nutrients and cannot fully represent the coupling of plant-soil nutrients. It is recommended to add indicators such as litter and improve relevant data;

2. It is recommended to systematically review the existing research gaps and precisely highlight the unique innovations of this study;

3. This study focuses on the chemometric succession patterns of soil carbon and nitrogen during forest restoration. However, the article only uses "Yunnan pine forest → mixed coniferous and broad-leaved forest → evergreen broad-leaved forest" to represent the restoration time gradient, without comparing the chronological gradient of plots, which presents limitations;

4. For the discussion section, delete discussions on relevant data that were not studied in this research. For example, this article does not include data on microorganisms or enzymes, yet the discussion section cites discussions on related mechanisms such as microbial decomposition. It is recommended to add comparative discussions with other areas in the discussion section to clarify the differentiated conclusions of this research.

**********

what does this mean?). If published, this will include your full peer review and any attached files.

If you choose “no”, your identity will remain anonymous but your review may still be made public.

**Do you want your identity to be public for this peer review?**  For information about this choice, including consent withdrawal, please see our Privacy Policy

Reviewer #1: No

Reviewer #2: No

**********

[NOTE: If reviewer comments were submitted as an attachment file, they will be attached to this email and accessible via the submission site. Please log into your account, locate the manuscript record, and check for the action link "View Attachments". If this link does not appear, there are no attachment files.]

To ensure your figures meet our technical requirements, please review our figure guidelines: https://journals.plos.org/plosone/s/figures

You may also use PLOS’s free figure tool, NAAS, to help you prepare publication quality figures: https://journals.plos.org/plosone/s/figures#loc-tools-for-figure-preparation.

NAAS will assess whether your figures meet our technical requirements by comparing each figure against our figure specifications.

# Author comment: Author response to Decision Letter 1

**DOI:** 10.1371/journal.pone.0356261.r002

9 Jul 2026

Academic Editor, Concern # 1: Please ensure that your manuscript meets PLOS ONE's style requirements, including those for file naming.

Author response: We have carefully followed PLOS ONE’s style templates and ensured that all submitted files—including file names—meet the journal’s requirements. Please let us know if any further adjustments are needed.

Academic Editor, Concern # 2: Please note that PLOS One has specific guidelines on code sharing for submissions in which author-generated code underpins the findings in the manuscript. In these cases, we expect all author-generated code to be made available without restrictions upon publication of the work. Please review our guidelines and ensure that your code is shared in a way that follows best practice and facilitates reproducibility and reuse.

Author response: We have read the code sharing guidelines carefully and have included all data and author-generated code as supplementary files submitted together with the manuscript. Please let us know if any further revisions are needed.

Academic Editor, Concern # 3: In your Methods section, please provide additional information regarding the permits you obtained for the work. Please ensure you have included the full name of the authority that approved the field site access and, if no permits were required, a brief statement explaining why.

Author response: We thank the editor for raising this point. In the revised manuscript, we have added a clear statement in the Methods section to explain that no specific permits were required for our field work, as the study sites are openly accessible and involve only non destructive sampling.

Author action: At the end of Section 2.1 (Profile of the Study Area), we have inserted the following sentence : The study sites are located in collectively owned general forestlands with no protected area designation. They are openly accessible without administrative restrictions, and sampling involved only non destructive collection of soil and leaves.

Academic Editor, Concern # 4: When completing the data availability statement of the submission form, you indicated that you will make your data available on acceptance. We strongly recommend all authors decide on a data sharing plan before acceptance, as the process can be lengthy and hold up publication timelines. Please note that, though access restrictions are acceptable now, your entire data will need to be made freely accessible if your manuscript is accepted for publication. This policy applies to all data except where public deposition would breach compliance with the protocol approved by your research ethics board. If you are unable to adhere to our open data policy, please kindly revise your statement to explain your reasoning and we will seek the editor's input on an exemption. Please be assured that, once you have provided your new statement, the assessment of your exemption will not hold up the peer review process.

Author response: We have discussed the data sharing plan among all co-authors and fully agree to make all data and code available upon publication, in line with PLOS ONE’s policy. We will revise the Data Availability Statement accordingly and do not request any exemption. Please let us know if further changes are needed.

Academic Editor, Concern # 5: PLOS requires an ORCID iD for the corresponding author in Editorial Manager on papers submitted after December 6th, 2016. Please ensure that you have an ORCID iD and that it is validated in Editorial Manager.

Author response: We confirm that all authors have valid ORCID iDs and that they have been successfully validated in Editorial Manager.

Academic Editor, Concern # 6: Please ensure that you refer to Figures 1 - 4 in your text as, if accepted, production will need this reference to link the reader to the figure.

Author response: We have reviewed the manuscript and added explicit in text citations for Figures 1–4 in the appropriate places to ensure proper linking during production. Please let us know if further changes are required.

Academic Editor, Concern # 7: We note that Figure 1 in your submission contain map images which may be copyrighted. All PLOS content is published under the Creative Commons Attribution License (CC BY 4.0), which means that the manuscript, images, and Supporting Information files will be freely available online, and any third party is permitted to access, download, copy, distribute, and use these materials in any way, even commercially, with proper attribution. For these reasons, we cannot publish previously copyrighted maps or satellite images created using proprietary data, such as Google software (Google Maps, Street View, and Earth).

Author response: We appreciate the editor’s concern regarding the copyright of Figure 1. We confirm that the base map for Figure 1 was obtained from the Geospatial Data Cloud (http://www.gscloud.cn), operated by the Computer Network Information Center, Chinese Academy of Sciences, and is not derived from any proprietary or copyrighted sources such as Google Maps or other commercial software. We have also attached a screenshot of the data usage and copyright statement from the website as supporting evidence for your reference. We trust this addresses the copyright concern. Please let us know if further modifications are required.

Author action: In the revised manuscript, we have already added a clear data source citation in the figure caption: “The data set is provided by Geospatial Data Cloud site, Computer Network Information Center, Chinese Academy of Sciences (http://www.gscloud.cn).”

Academic Editor, Concern # 8: If the reviewer comments include a recommendation to cite specific previously published works, please review and evaluate these publications to determine whether they are relevant and should be cited. There is no requirement to cite these works unless the editor has indicated otherwise.

Author response: We sincerely thank the editor for this suggestion. We have carefully reviewed the five papers recommended by Reviewer 1. One of them (Li et al., 2025) is directly relevant to our study and has been cited in the revised manuscript. The remaining four papers, while not directly overlapping with our specific focus, were nonetheless carefully read and offered useful conceptual perspectives. Please let us know if any further modifications are required.

Reviewer#1, Concern # 1: The study includes only three replicate regions per forest type, and soil samples from five points within each plot were composited into a single sample. Please clarify whether the true sample size for statistical analyses was n = 3 or n = 27. The potential issue of pseudoreplication should be discussed because it may inflate statistical significance.

Author response: We sincerely thank the reviewer for raising this important point. We honestly acknowledge that this was a writing error in our initial manuscript. In the actual field design, we established nine independent 20 m × 20 m plots per forest type (three on the upper slope, three on the middle slope, and three on the lower slope), and all statistical analyses—including RDA, Random Forest, and Mantel tests—were performed based on the true data from these nine plots (i.e., n=9 per forest type). The five-point composite sampling was employed solely as a technical procedure to reduce within-plot micro-variability and was never treated as statistical replication. In the revised manuscript, we have corrected the misleading description from "three regions" to "nine plots" in the Methods section, and explicitly defined the plot as the true independent unit for statistical testing in both the Methods and results sections. Therefore, our statistical inferences are firmly based on genuine independent plots, and the concern about pseudoreplication inflating statistical significance does not apply to our study. We apologize for any confusion caused by this descriptive error.

Author action:We have corrected the replicate description in Section 2.2.1 by replacing the entire erroneous paragraph with a revised version that changes "three regions" to "nine plots" and updates the sample numbers from 27 to 81 composite samples and from 81 to 243 undisturbed cores. A clarifying statement has also been added within this section to emphasize that the plot (n=9 per forest type) is the true independent unit for statistical analyses. Additionally, in Section 3.2, we have supplemented the beginning of the RDA results with a sentence specifying that the analysis was based on plot-level replicates. All corresponding changes have been highlighted in the revised manuscript.

Reviewer#2, Concern # 2: The study interprets differences among forest types as restoration-stage effects. However, the manuscript does not provide evidence that the three forest types represent a true successional sequence with similar historical land-use conditions. More justification is needed to support this assumption.

Author response: We sincerely thank the reviewer for this critical and constructive comment. We fully agree that the assumption of a true successional sequence with comparable historical and environmental backgrounds must be rigorously justified when employing a space-for-time substitution approach. In response, we have undertaken three complementary modifications to strengthen this rationale. First, in the Introduction (Section 1), we added literature citations in the Introduction to establish the regional representativeness of this successional trajectory. Second, in Materials and Methods (Section 2.2.1), we have added quantitative stand structural parameters—specifically, mean tree height and diameter at breast height (DBH)—which exhibit clear increasing gradients across the three forest types, providing direct empirical evidence that these communities represent structurally distinct and progressively more advanced restoration stages. Third, in the Discussion (Section 4), we have included a cautious acknowledgment of the inherent limitations of the space-for-time substitution approach, while also clarifying that our plot selection minimized variations in topography, soil parent material, and climatic background, thereby supporting the inference that observed differences primarily reflect restoration progression rather than inherent site heterogeneity.

Author action: 1. In the Introduction (Section 1), we added the following text: In the study region, previous phytosociological studies have demonstrated that secondary succession following anthropogenic disturbance typically follows a predictable trajectory from pioneer Pinus yunnanensis forests, through mixed conifer–broadleaf transitional communities, towards the zonal semi-humid evergreen broadleaved forests[46]. This well-documented successional framework provides a robust and ideal chronosequence for examining how plant–soil nutrient associations and soil stoichiometric regulation evolve during ecosystem recovery. Moreover, the successional classification of tree species utilized in local restoration trials further corroborates the functional differentiation of these seral stages, reinforcing their suitability for such analysis[47].

2.In Materials and Methods (Section 2.2.1), we inserted the following text after describing the plot establishment, together with a new Supplementary Table S1 containing detailed stand structural parameters: In addition, the three forest types exhibited clear structural gradients consistent with successional progression: the mean stand height and diameter at breast height (DBH) increased progressively from the Pinus yunnanensis forest (height: 10.9 ± 0.9 m; DBH: 87.3 ± 2.8 mm) through the mixed conifer–broadleaf forest (height: 11.1 ± 0.9 m; DBH: 92.5 ± 7.6 mm) to the evergreen broadleaf forest (height: 11.6 ± 0.6 m; DBH: 96.1 ± 10.8 mm), further supporting their designation as distinct restoration stages (detailed stand structural parameters are provided in Table S1).

3.In the Discussion (Section 4), we added the following paragraph at the end to acknowledge methodological limitations while reinforcing the validity of our approach: We acknowledge that soil sampling was confined to the single peak growing season (June 2025) and thus did not capture seasonal variations in nutrient availability. Nevertheless, sampling during this biologically active period provides a consistent baseline for comparing restoration-driven changes across forest types, and our interpretations are strictly confined to static chemical associations and physico-chemical equilibria, as microbial biomass and enzymatic activities were not measured. Future multi-seasonal monitoring, ideally combined with microbiological and isotopic approaches, is needed to fully disentangle the temporal and biotic dimensions of stoichiometric dynamics.

Reviewer#1, Concern # 3: Soil samples were collected only in June 2025, while plant samples were collected in June and November 2025. Seasonal and interannual variability can strongly affect nutrient availability and stoichiometry. The limitation of single-year sampling should be clearly acknowledged.

Author response: We sincerely thank the reviewer for this valuable comment. We fully agree that seasonal and interannual variability can affect soil nutrient availability, and we acknowledge this limitation explicitly. Accordingly, we have added a concise paragraph at the end of the Discussion section to address this issue, while also providing a brief justification for our sampling strategy during the peak growing season.

Author action: We added one short paragraph at the end of the Discussion section to acknowledge that soil sampling was confined to June 2025 and did not capture seasonal variations, while noting that the peak growing season provides a consistent baseline for comparing restoration stages and calling for future multi-seasonal monitoring.

Added text: We acknowledge that soil sampling was confined to the single peak growing season (June 2025) and thus did not capture seasonal variations in nutrient availability. Nevertheless, sampling during this biologically active period provides a consistent baseline for comparing restoration-driven changes across forest types, and our interpretations are strictly confined to static chemical associations and physico-chemical equilibria, as microbial biomass and enzymatic activities were not measured. Future multi-seasonal monitoring, ideally combined with microbiological and isotopic approaches, is needed to fully disentangle the temporal and biotic dimensions of stoichiometric dynamics.

Reviewer#1, Concern # 4: The RDA results report explained variances of 99.11% and 99.62% for the mixed forest and evergreen broadleaf forest, respectively. Such extremely high explanatory power is unusual in ecological field studies and may indicate overfitting or multicollinearity among explanatory variables. The authors should verify these results and provide variance inflation factor (VIF) analysis.

Author response: We sincerely thank the reviewer for this rigorous and highly constructive statistical comment.To rigorously address the potential overfitting and multicollinearity issues, we implemented a strict Variance Inflation Factor (VIF) screening procedure. Explanatory variables with VIF > 10 were sequentially removed from the models to eliminate redundant collinearity(the detailed VIF values and screening results are now provided in Table S1 of the Supplementary Materials).

Reviewer#1, Concern # 5: The Random Forest model uses soil C, C, and N ratios as predictor variables when explaining other stoichiometric ratios. This may introduce circular reasoning because response variables are mathematically related to predictor variables. The modeling strategy should be reconsidered and justified.

Author response: We sincerely thank the reviewer for identifying this critical statistical issue. We fully agree that using mathematically interrelated ratio variables (e.g., using C:N and N:P to predict C:P) introduces circularity and may obscure true ecological mechanisms. Following the reviewer's suggestion, we have thoroughly reconsidered and reconstructed our modeling strategy, re-ran all relevant analyses, and systematically revised the corresponding texts in the Results, Discussion, Conclusion, a

Attachment Submitted filename: Response to Reviewers.docx

# Peer review: Decision Letter 1

**DOI:** 10.1371/journal.pone.0356261.r003

**Authors:** Jian Liu

3 Aug 2026

Successional Shifts in Soil C:N:P Stoichiometry and Its Environmental Controls During Forest Restoration in Huize County, Qujing, Yunnan Province

PONE-D-26-28435R1

Dear Dr. Hu,

We’re pleased to inform you that your manuscript has been judged scientifically suitable for publication and will be formally accepted for publication once it meets all outstanding technical requirements.

Within one week, you’ll receive an e-mail detailing the required amendments. When these have been addressed, you’ll receive a formal acceptance letter and your manuscript will be scheduled for publication.

An invoice will be generated when your article is formally accepted. Please note, if your institution has a publishing partnership with PLOS and your article meets the relevant criteria, all or part of your publication costs will be covered. Please make sure your user information is up-to-date by logging into Editorial Manager at Editorial Manager® and clicking the ‘Update My Information' link at the top of the page. For questions related to billing, please contact billing support.

If your institution or institutions have a press office, please notify them about your upcoming paper to help maximize its impact. If they’ll be preparing press materials, please inform our press team as soon as possible -- no later than 48 hours after receiving the formal acceptance. Your manuscript will remain under strict press embargo until 2 pm Eastern Time on the date of publication. For more information, please contact onepress@plos.org.

Kind regards,

Jian Liu

Academic Editor

PLOS One

Additional Editor Comments (optional):

All comments have been addressed.

Reviewers' comments:

Reviewer's Responses to Questions

**Comments to the Author**

Reviewer #1: All comments have been addressed

Reviewer #2: All comments have been addressed

**********

2. Is the manuscript technically sound, and do the data support the conclusions?

Reviewer #1: Yes

Reviewer #2: Yes

**********

3. Has the statistical analysis been performed appropriately and rigorously?

Reviewer #1: Yes

Reviewer #2: Yes

**********

4. Have the authors made all data underlying the findings in their manuscript fully available?

The PLOS Data policy

Reviewer #1: Yes

Reviewer #2: Yes

**********

5. Is the manuscript presented in an intelligible fashion and written in standard English?

Reviewer #1: Yes

Reviewer #2: Yes

**********

Reviewer #1: The authors have adequately addressed all of my comments and incorporated the suggested revisions. I have no further comments on the manuscript. Therefore, I recommend that the manuscript be accepted for publication.

Reviewer #2: (No Response)

**********

what does this mean?). If published, this will include your full peer review and any attached files.

If you choose “no”, your identity will remain anonymous but your review may still be made public.

**Do you want your identity to be public for this peer review?**  For information about this choice, including consent withdrawal, please see our Privacy Policy

Reviewer #1: No

Reviewer #2: No

**********

# Editor report: Acceptance letter

**DOI:** 10.1371/journal.pone.0356261.r004

**Authors:** Jian Liu

PONE-D-26-28435R1

PLOS One

Dear Dr. Hu,

I'm pleased to inform you that your manuscript has been deemed suitable for publication in PLOS One. Congratulations! Your manuscript is now being handed over to our production team.

At this stage, our production department will prepare your paper for publication. This includes ensuring the following:

* All references, tables, and figures are properly cited

* All relevant supporting information is included in the manuscript submission,

* There are no issues that prevent the paper from being properly typeset

You will receive further instructions from the production team, including instructions on how to review your proof when it is ready. Please keep in mind that we are working through a large volume of accepted articles, so please give us a few days to review your paper and let you know the next and final steps.

Lastly, if your institution or institutions have a press office, please let them know about your upcoming paper now to help maximize its impact. If they'll be preparing press materials, please inform our press team within the next 48 hours. Your manuscript will remain under strict press embargo until 2 pm Eastern Time on the date of publication. For more information, please contact onepress@plos.org.

You will receive an invoice from PLOS for your publication fee after your manuscript has reached the completed accept phase. If you receive an email requesting payment before acceptance or for any other service, this may be a phishing scheme. Learn how to identify phishing emails and protect your accounts at https://explore.plos.org/phishing.

If we can help with anything else, please email us at customercare@plos.org.

Thank you for submitting your work to PLOS ONE and supporting open access.

Kind regards,

PLOS ONE Editorial Office Staff

on behalf of

Dr. Jian Liu

Academic Editor

PLOS One
